# Extrachromosomal circular DNA promotes inflammation and hepatocellular carcinoma development

**DOI:** 10.1126/sciadv.adw0272

**Published:** 2025-10-17

**Authors:** Lap Kwan Chan, Juanjuan Shan, Elias Rodriguez-Fos, Marc Eamonn Healy, Peter Leary, Rossella Parrotta, Nina Desboeufs, Gabriel Semere, Nadine Wittstruck, Anton G. Henssen, Achim Weber

**Affiliations:** ^1^ Department of Pathology and Molecular Pathology, University Hospital of Zurich, Zurich, Switzerland.; ^2^ Institute of Molecular Cancer Research, University of Zurich, Zurich, Switzerland.; ^3^ Chongqing Key Laboratory of Translational Research for Cancer Metastasis and Individualized Treatment, Chongqing University Cancer Hospital, Chongqing, China.; ^4^ Department of Pediatric Oncology/Hematology, Charité-Universitätsmedizin Berlin, Berlin, Germany.; ^5^ Functional Genomics Center Zurich, University of Zurich/ETHZ, Zurich, Switzerland.

## Abstract

Two decades after the initial report on increased micronuclei in human chronic liver disease (CLD) and hepatocellular carcinoma (HCC), their role in HCC development is still poorly understood. Here, we show that micronuclei in hepatocytes trigger a hepatic immune response and promote HCC development via an increased level of extrachromosomal circular DNA (eccDNA). Livers of a CLD model (*Mcl1*^Δhep^ mice) show increased micronuclei and eccDNA levels. Circular sequencing confirms higher eccDNA levels in micronuclei compared to primary nuclei. The nuclei-segregated DNA fiber (NuSeF) assay we developed demonstrates that micronuclei are more susceptible to replication stress, exhibiting increased replication fork slowing. Comparing different murine liver disease models reveals that high eccDNA correlates with an increased tumor incidence. eccDNA is a strong immunostimulant and promotes a cross-talk between hepatocytes and immune cells through the cGAS-STING pathway. Deletion of *Sting1* in *Mcl1*^Δhep^ mice reduces immune cell chemotaxis and tumor incidence. Our findings suggest that eccDNA from micronuclei mediates inflammation-driven liver carcinogenesis in CLD.

## Teaser

Replication defects in micronuclei contribute to the generation of eccDNA and consequently promote inflammation in CLD.

## INTRODUCTION

In 2020, primary liver cancer ranked as the sixth most common type of cancer and the third leading cause of cancer-related deaths (*1*). The number of newly diagnosed liver cancers is expected to increase over the next two decades, reaching 1.4 million in 2040. Hepatocellular carcinoma (HCC) is the most prevalent type of liver cancer and accounts for more than 80% of all primary liver cancers (*2*). Most HCCs develop as a result of a long-term chronic liver disease (CLD). In recent years, the incidence of a particular CLD, namely metabolic dysfunction-associated steatotic liver diseases, has risen markedly worldwide (*3*, *4*). Chronic liver inflammation and fibrosis, which are associated with constant liver cell death, can subsequently progress to cirrhosis and markedly increase the risk of HCC development. Hepatocyte hyperproliferation is a phenomenon frequently observed in CLD as a compensatory regenerative mechanism triggered by increased cell death and supported by a pro-inflammatory microenvironment (*5*, *6*). The production of immunogenic molecules, known as damage-associated molecular patterns, from damaged cells is responsible for triggering immune responses and sustains the status of “sterile inflammation” (*7*). Under such pro-inflammatory microenvironments, hepatocytes can experience increased DNA damage and genetic instability, which can eventually promote liver carcinogenesis (*8*).

Myeloid cell leukemia 1 (MCL1) is a prosurvival member of the B cell lymphoma 2 (Bcl-2) family, which shares BH domains with Bcl-2 and Bcl-X_L_ (*9*). MCL1 inhibits apoptosis by sequestering the proapoptotic proteins Bak (Bcl-2 homologous antagonist killer) and Bax (Bcl-2–associated protein X), thus counteracting mitochondrial outer membrane permeabilization during the initiation of apoptosis. Our group previously reported that liver-specific knockout of *Mcl1* in mice (*Mcl1*^Δhep^) resulted in a phenotype resembling CLD in patients characterized by elevated apoptosis, compensatory proliferation, and DNA damage, with increased tumor incidence at 12 months (*6*, *8*). These observations suggested that the increase in DNA damage and genomic instability in hepatocytes in particular are directly linked to carcinogenesis. However, it is still unclear whether the carcinogenesis from CLD to HCC is mediated by a cell-autonomous or non–cell-autonomous mechanism. In the current study, we use *Mcl1*^Δhep^ mice as a CLD model and find an important mechanism involving a cross-talk between hepatocytes and immune cells in the liver. This is mediated by extrachromosomal circular DNA (eccDNA) and driven by a pro-inflammatory microenvironment in a manner that is dependent on the cyclic GMP-AMP synthase (cGAS)-stimulator of interferon genes (STING) signaling pathway. Our findings suggested that a non–cell-autonomous mechanism is involved in liver tumorigenesis, with micronuclei (MN) and eccDNA playing a pivotal role. Moreover, we present a previously not implemented method that allows us to study replication stress within primary nuclei (PN) and MN, thus providing mechanistic insights into the formation of eccDNA.

## RESULTS

### Livers of *Mcl1*^Δhep^ mice show increased MN levels and an activated cGAS-STING pathway

**Fig. 1.**
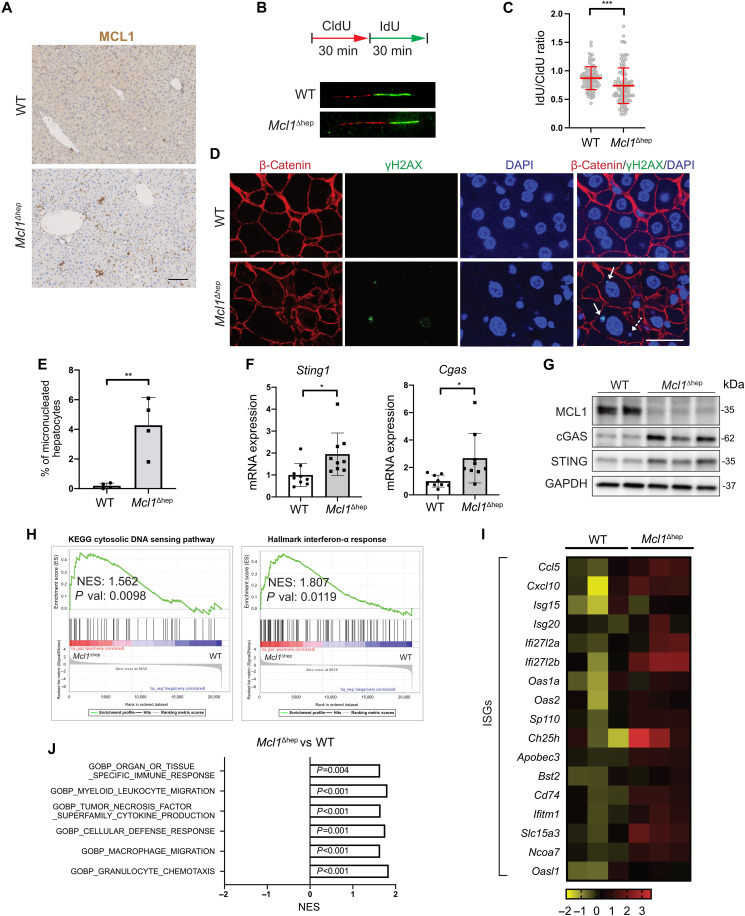
Deletion of MCL1 in hepatocytes leads to increased micronucleated hepatocytes and activation of the cGAS-STING pathway. (**A**) MCL1 immunohistochemistry staining on WT and *Mcl1*^Δhep^ livers. Scale bar, 100 μm. (**B**) DNA fiber assay on primary hepatocytes from WT and *Mcl1*^Δhep^ mice after 24 hours in culture. (**C**) Quantification of IdU-to-CldU ratio. (**D**) Immunofluorescence staining of 2-month-old liver tissue showing the presence of γH2AX^+^ (arrow) and γH2AX^−^ MN (dashed arrow) in hepatocytes. Scale bar, 25 μm. DAPI, 4′,6-diamidino-2-phenylindole. (**E**) Quantification of micronucleated hepatocytes as a percentage of the total hepatocytes. *n* = 4. (**F**) Quantitative polymerase chain reaction (PCR) showing the up-regulation of the cGAS and STING expression in the *Mcl1*^Δhep^ liver. *n* = 9. (**G**) Immunoblot of protein lysates from 2-month-old WT and *Mcl1*^Δhep^ livers showing the up-regulation of the cGAS and STING protein levels. GAPDH, glyceraldehyde-3-phosphate dehydrogenase. (**H**) GSEA showing an enrichment of the cytosolic DNA sensing pathway and the interferon-α response in *Mcl1*^Δhep^ livers. NES, net enrichment score; KEGG, Kyoto Encyclopedia of Genes and Genomes. (**I**) Heatmap showing the up-regulation of ISGs in *Mcl1*^Δhep^ livers. (**J**) *Mcl1*^Δhep^ mice showed a significant enrichment of various gene signatures involved in immune responses and immune cell recruitment. Student’s *t* test: *P < 0.05, **P < 0.01, and ***P < 0.001.

Previously, we reported that *Mcl1*^Δhep^ mice exhibited liver hyperproliferation already at 2 months of age (*6*, *8*). To determine whether the observed increase in DNA damage in *Mcl1*^Δhep^ livers was a consequence of increased replication stress, we performed DNA fiber assay on primary hepatocytes from wild-type (WT) and *Mcl1*^Δhep^ mice. We established MCL1 immunohistochemistry to confirm a high knockout efficiency and observed that MCL1 expression was almost restricted to nonparenchymal cells (NPCs) in *Mcl1*^Δhep^ livers (Fig. 1A). Pulse labeling of two thymidine analogs, 5-chloro-2′-deoxyuridine (CldU) and 5′-iodo-2′-deoxyuridine (IdU), allowed the monitoring of the replication fork dynamics and detection of replication stress in cells (*10*, *11*). We observed that MCL1-deficient hepatocytes displayed a significantly longer CldU tract length with no difference in the total tract length (CldU + IdU) (Fig. 1B and fig. S1A). The ratio of IdU to CldU, however, was significantly reduced in *Mcl1*^Δhep^ hepatocytes compared to WT (Fig. 1C). This suggested that *Mcl1*^Δhep^ hepatocytes had a higher rate of replication fork stalling, a sign of increased replication stress. Given that the formation of MN had been associated with increased DNA damage and replication stress (*12*), we examined the level of micronucleated hepatocytes in *Mcl1*^Δhep^ livers. Using β-catenin as a membrane marker, we visualized and found a significantly higher level of micronucleated hepatocytes in the *Mcl1*^Δhep^ livers compared to WT livers (Fig. 1, D and E; fig. S1, B and C; and table S1). These results suggested that MCL1 deficiency in hepatocytes caused a higher replication stress and led to increased MN formation.

The cytosolic DNA sensing pathway was recently reported to connect DNA damage to the activation of IRF3 (interferon regulatory factor 3) and nuclear factor κB (NF-κB), both known as important transcription factors for immune response (*13*). We therefore analyzed whether this pathway was activated in the *Mcl1*^Δhep^ liver. The expression of the key components of this pathway, cGAS and STING, was up-regulated in 2- and 12-month-old *Mcl1*^Δhep^ livers at both mRNA and protein levels (Fig. 1, F and G, and fig. S1D). Transcriptomic analysis of 2-month-old liver samples showed an enrichment of the cytosolic DNA sensing pathway and the interferon-α response signatures in the *Mcl1*^Δhep^ liver (Fig. 1H). Activation of the cGAS-STING pathway is known to facilitate the production of various interferon-stimulated genes (ISGs) (*12*). Analysis of an ISG list showed that these genes were up-regulated in the 2-month-old *Mcl1*^Δhep^ liver, including genes encoding chemokines (*Ccl5* and *Cxcl10*) and interferon-α–inducible genes (*Ifi27l2a* and *Ifi27l2b*) (Fig. 1I). Several immune response–related signatures, such as pathways promoting immune cell chemotaxis, were also enriched in *Mcl1*^Δhep^ livers (Fig. 1J). To corroborate these results, we performed immunohistochemistry and found that the infiltration of B cells (B220^+^), neutrophils (Ly6G^+^), and macrophages (F4/80^+^) was significantly higher in *Mcl1*^Δhep^ livers (fig. S2, A and B).

Thus, an activation of the cGAS-STING pathway was observed in *Mcl1*^Δhep^ livers, even though hepatocytes had been shown to have an incomplete cGAS-STING pathway (*14*–*16*). Using immunohistochemical staining, we observed that both hepatocytes and NPCs expressed cGAS (fig. S2C). Expression of STING, however, was restricted to NPCs (fig. S2, C and D). Taking advantage of serial sections, we visualized a colocalization of F4/80^+^ and STING (fig. S2E). This pattern was further confirmed in isolated immune cells and hepatocytes using Western blotting (fig. S2, F and G). Some of the cGAS^+^ hepatocytes appeared to express a proliferation marker (Ki67) or DNA damage marker [phosphorylated H2AX at S139 (γH2AX)] (fig. S2H). Because of the differences in the spatial expression pattern of cGAS and STING, we sought to determine whether there is a mechanism mediating a cross-talk between DNA damage in hepatocytes and the activation of the cGAS-STING pathway in immune cells. One of the reported mechanisms to promote a cross-talk between cGAS-activated cells and adjacent bystander cells is via the production and transfer of the second messenger molecule cyclic guanosine monophosphate-adenosine monophosphate (cGAMP) (*17*, *18*). Measurement of cGAMP using the enzyme-linked immunosorbent assay showed that there was no significant change in the cGAMP level between WT and *Mcl1*^Δhep^ liver lysates (fig. S2I). Therefore, presumably, other mechanisms are involved in facilitating the cross-talk between hepatocytes and immune cells in the *Mcl1*^Δhep^ liver.

### eccDNA is increased in *Mcl1*^Δhep^ livers and has a strong immunostimulatory activity

**Fig. 2.**
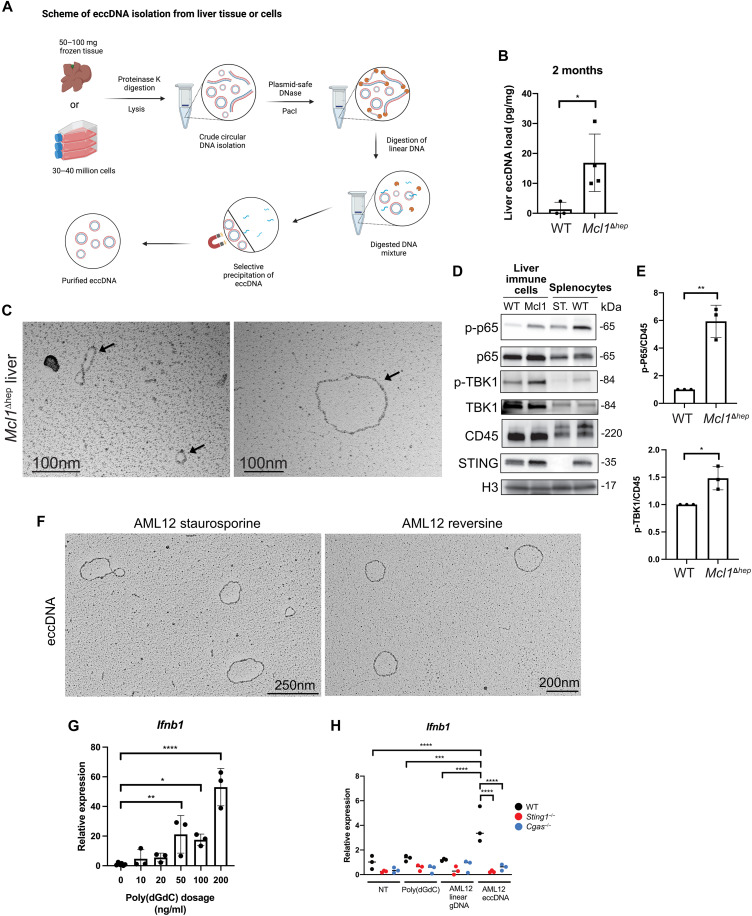
The cross-talk between MCL1-deficient hepatocytes and immune cells is mediated through an increase in eccDNA levels. (**A**) Schematic representation showing the steps of eccDNA isolation from liver tissues and cultured cells. Created in BioRender. L. K. Chan (2025); https://biorender.com/wqbnjku. DNase, deoxyribonuclease. (**B**) Quantification of eccDNA isolated from WT and *Mcl1*^Δhep^ livers by the Qubit HS dsDNA kit (pg/mg of tissues). Student’s *t* test: *P < 0.05. WT: *n* = 3; *Mcl1*^Δhep^: *n* = 4. (**C**) Visualization of eccDNA (arrow) isolated from the *Mcl1*^Δhep^ liver by electron microscopy (pictures show eccDNA captured from different fields). Scale bar, 100 nm. (**D**) Immune cells isolated from the *Mcl1*^Δhep^ liver showing higher p-p65 and STING levels. Mcl1, *Mcl1*^Δhep^; ST, *Sting1*^−/−^. (**E**) Densitometric analysis of Western blot for p-p65 and p-TBK1 levels normalized to the CD45 level from three independent experiments as a fold change compared to WT. Student’s *t* test: *P < 0.05 and **P < 0.01. (**F**) Visualization of eccDNA isolated from AML12 cells treated with 0.5 μM staurosporine for 24 hours or 0.5 μM reversine for 48 hours. Scale bars, 250 and 200 nm. (**G**) Induction of *Ifnb1* expression analyzed by quantitative PCR after poly(dGdC) treatment on WT BMDMs showed a dose-dependent pattern. Student’s *t* test: *P < 0.05. *n* = 3. (**H**) BMDMs from WT, *Sting1*^−/−^, and *Cgas*^−/−^ mice were transfected with eccDNA (10 ng/ml), linear genomic DNA (10 ng/ml), and poly(dGdC) (10 ng/ml). The *Ifnb1* expression was analyzed 12 hours after the transfection. *n* = 3. One-way ANOVA: ***P < 0.001 and ****P < 0.0001.

eccDNA is a class of circular DNA elements that can be found in different tissues (*19*). The biogenesis of eccDNA is still not completely understood, although recent evidence showed that their generation might be related to DNA damage, increased cellular stress, and apoptosis (*20*, *21*). We performed eccDNA isolation using a protocol established for cultured cells and adapted it for liver tissue (Fig. 2A) (*22*). We observed that 2-month-old *Mcl1*^Δhep^ livers contained more eccDNA compared to age-matched WT controls (Fig. 2B). Using electron microscopy, we were able to visualize and confirm the presence of eccDNA using a plasmid (pRSV) as a control (Fig. 2C and fig. S3, A and B). To test our hypothesis that the observed activation of the cGAS-STING pathway in *Mcl1*^Δhep^ livers was due to the activation in immune cells, we isolated immune cells and examined their STING and p-p65 levels. Immune cells from *Mcl1*^Δhep^ livers showed a stronger STING expression and higher NF-κB activities, as indicated by increased phosphorylation of p65 at S536 (*23*), compared to immune cells from WT livers (Fig. 2, D and E, and fig. S3C). Analysis of the downstream kinase of STING, TBK1 (TANK-binding kinase 1), also showed an increased level of phosphorylation at S172 in the immune cells from *Mcl1*^Δhep^ livers (Fig. 2, D and E, and fig. S3C). These observations were consistent with the observed activation of the cGAS-STING pathway in *Mcl1*^Δhep^ livers.

eccDNA from HeLa cells has been shown to be a strong immunostimulant (*20*). Therefore, we tested the effect of eccDNA from hepatocytes on immune cells and compared them with linear DNA and poly(deoxyguanylic-deoxycytidylic) acid [poly(dGdC)]. To ensure that an adequate amount of eccDNA was generated in hepatocytes, we treated AML12 cells, a murine hepatocyte cell line, with staurosporine using HeLa cells in parallel as a control. Treatment with staurosporine for 24 hours strongly increased the percentage of apoptotic cells in AML12 and HeLa cells (fig. S3, D and E). eccDNA from these cells was isolated and visualized by electron microscopy (Fig. 2F and fig. S3F). Similarly, treatment of AML12 cells over 48 hours with reversine, a reagent known to induce lagging chromosomes and MN formation, also resulted in eccDNA generation (Fig. 2F). Of note, reversine treatment did not induce extensive apoptosis in AML12 cells as staurosporine treatment did. To compare the immune-stimulating potency of eccDNA with linear DNA on immune cells, we generated bone marrow–derived macrophages (BMDMs) from 2-month-old mice of different genotypes (fig. S3, G and H). Poly(dGdC) is a synthetic DNA construct and a known potent agonist of STING. We tested the ability of poly(dGdC) to induce *Ifnb1* expression in WT BMDMs and found a threshold-dependent positive association between the dose of poly(dGdC) and *Ifnb1* induction (Fig. 2G). At a dosage in which both poly(dGdC) and linear DNA were unable to induce *Ifnb1* expression in WT BMDMs, eccDNA from AML12 cells induced a significantly higher expression of *Ifnb1* and *Tnfa* (Fig. 2H and fig. S3I). A similar result was observed with HeLa cell eccDNA, which induced a much stronger expression of *Ifnb1* and *Tnfa* in WT BMDMs (fig. S3J). Such induction was strongly dependent on the cGAS-STING pathway, as a deletion of either cGAS or STING abolished *Ifnb1* and *Tnfa* inductions by eccDNA (Fig. 2H and fig. S3, I and J). These results confirmed that eccDNA had strong immunostimulating properties, and their activities were dependent on both cGAS and STING.

It has been shown that DNA with different sizes has different potency in activating the cGAS-STING pathway with small cytosolic dsDNA [double-stranded DNA; ~20-40 base pairs (bp)] even having an inhibitory activity (*24*, *25*). We compared the DNA size in different categories used for BMDM stimulation. We observed that the majority of the eccDNA from AML12 and HeLa cells was approximately from 400 to 5000 bp in length, with discrete bands observed at lower molecular sizes (fig. S4A, lanes 3 and 5). Poly(dGdC), on the other hand, showed an enrichment of molecular size between 500 and 750 bp (fig. S4A, lane 1). Linear genomic DNA (sonicated) showed a DNA smear pattern, covering lengths from 250 bp to more than 10,000 bp. HeLa linear DNA was more abundant between 300 and 4000 bp (fig. S4A, lane 2). For AML12 linear DNA, we observed a higher abundance of DNA between 500 and 4000 bp and an enriched band at ~1200 bp (fig. S4A, lane 4). This result indicated that the sonicated genomic DNA samples [linear genomic DNA (gDNA)] from both cell lines not only contain DNA with sizes similar to the eccDNA samples but also DNA fragments larger than 5000 bp. To test whether the larger DNA fragments would contribute to the difference in their immunostimulating properties, we performed an additional sonication cycle, particularly on the AML12 linear gDNA sample, aiming to reduce the overall size of linear DNA, thus bringing their size closer to that of eccDNA. An additional sonication cycle reduced the enriched band at 1200 bp and enriched DNA with sizes around 250 to 2000 bp (fig. S4B). However, such a reduction in size did not affect the potency in inducing *Ifnb1* expression in BMDMs (fig. S4C). Collectively, this observation confirmed our finding that linear gDNA, in contrast to eccDNA, was not efficient in inducing *Ifnb1* expression in BMDMs, at least at low doses.

### CirSeq shows that *Mcl1*^Δhep^ livers compared to WT livers have a higher amount of eccDNA, which originates from all chromosomes

**Fig. 3.**
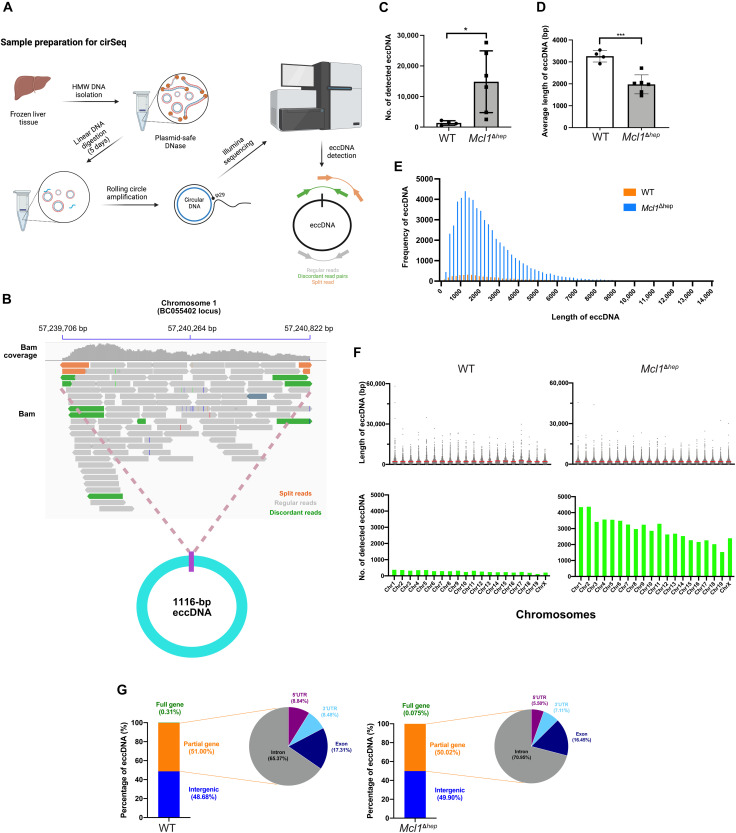
CirSeq shows that eccDNA from *Mcl1*^Δhep^ liver is shorter and comes from all chromosomes. (**A**) Schematic representation showing the steps of sample preparation and cirSeq. Created in BioRender. L. K. Chan (2025); https://biorender.com/w264fyb. HMW, high molecular weight. (**B**) Example of a 1116-bp eccDNA identified on chromosome 1 at the BC055402 locus of a *Mcl1*^Δhep^ liver sample. (**C**) Number of eccDNA detected in each liver sample. (**D**) Average length of eccDNA (base pairs) detected from each liver sample. WT: *n* = 4; *Mcl1*^Δhep^: *n* = 6. Student’s *t* test: *P < 0.05 and **P < 0.01. (**E**) Plotting of frequency of eccDNA according to different sizes (base pairs). Bin size: 200 bp. *n* = 4. (**F**) Size and number of detected eccDNA from each chromosome. Upper: Each dot represents one detected eccDNA. The red line indicates the mean. Lower: Data were summarized from four samples from each group. (**G**) The content of detected eccDNA was analyzed, and the percentages of full gene, partial gene, or intergenic region-containing eccDNA were shown. 3′UTR, 3′ untranslated region; 5′UTR, 5′ untranslated region.

To further study the characteristics of eccDNA from *Mcl1*^Δhep^ livers, we performed circular sequencing (cirSeq) using an established protocol (*26*). In contrast to the silica column–based extraction method, this protocol enabled the detection of both large and small circular DNA (*19*, *26*, *27*). Rolling circle amplification was performed on the purified circular DNA followed by 150-bp pair-ended Illumina sequencing (Fig. 3A). The average sequencing depth per sample was 10.01 million reads, and 99.76% of the reads were mapped to the reference genome. Via the identification of the regions with a high abundance of mapped reads, together with the presence of breakpoints, split reads, and outward-facing discordant read pairs, circular DNA structures could be identified. We were able to identify eccDNA of various sizes originating from different regions of the genome. For example, a 1116-bp eccDNA was found to originate from chr1:57,239,706 to 57,240,822 within the long noncoding RNA BC055402 locus of a *Mcl1*^Δhep^ liver sample (Fig. 3B). We detected an average of 1353 unique eccDNA in WT livers, but more than 10 times the amount in *Mcl1*^Δhep^ livers, with an average of 14,835 eccDNA per sample (Fig. 3C). This result was consistent with the observation using a different method reported above (Fig. 2B). Despite an increase in the number of eccDNA in *Mcl1*^Δhep^ samples, the average length of circular DNA was significantly shorter (Fig. 3D). We observed that the majority of the eccDNA had a length between 400 and 6000 bp, which accounted for 85.18% of eccDNA in the WT and 95.81% in the *Mcl1*^Δhep^ group, respectively (Fig. 3E). By aligning the sequences to each chromosome, we detected that eccDNA originated from all chromosomes, with the frequency correlating proportionally with the size of the individual chromosomes (Fig. 3F). Notably, there was no significant difference in the length of eccDNA originating from different chromosomes. Looking into the content of eccDNA from WT and *Mcl1*^Δhep^ livers, more than 99.6% of the eccDNA contained intergenic sequences or partial genes (Fig. 3G). eccDNA carrying partial genes from WT and *Mcl1*^Δhep^ samples originated mostly from intronic regions, although the level was slightly higher in the latter (65.37% versus 70.95%). In WT samples, 17 of 5413 detected eccDNA carried full genes (0.31%), while in *Mcl1*^Δhep^ samples, 44 of 58,860 eccDNA carried full genes (0.075%) (table S2). These results suggest that the increased eccDNA in the liver of *Mcl1*^Δhep^ mice consists mainly of smaller eccDNA originating from all chromosomes.

### MN contain more eccDNA compared to PN

**Fig. 4.**
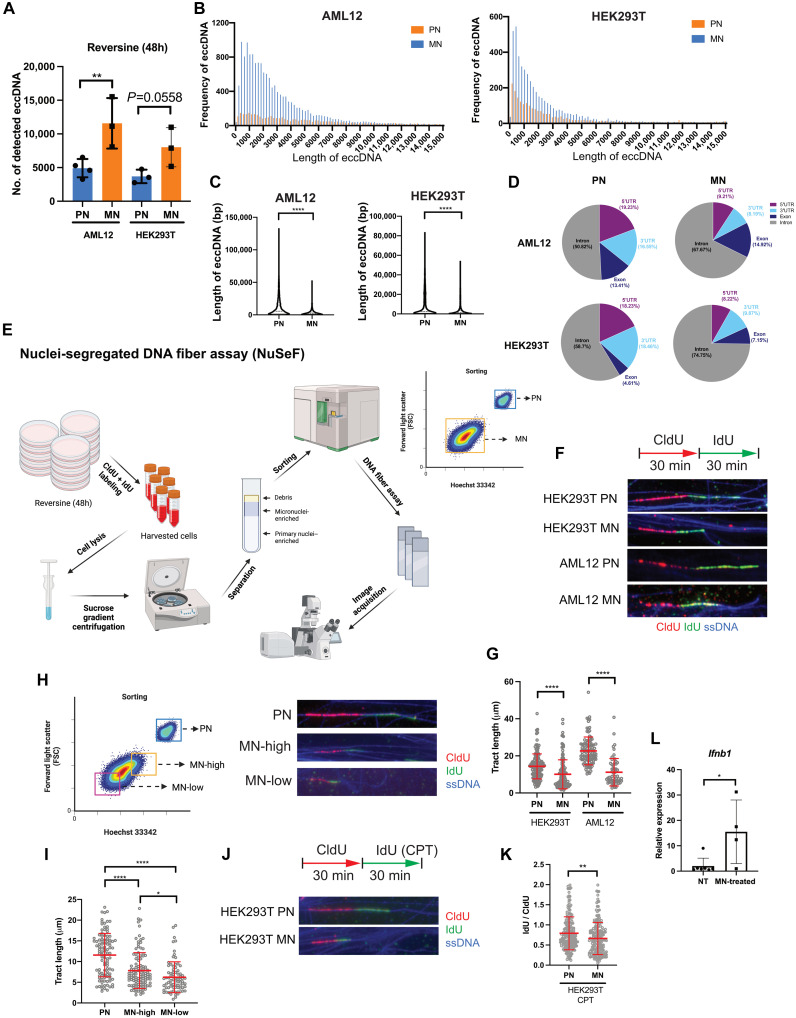
MN have more abundant eccDNA than PN. (**A**) CirSeq performed on sorted PN and MN. The number of detected eccDNA was compared. *n* ≥ 3. h, hours. (**B**) Plotting of frequency of eccDNA detected in AML12 and HEK293T PN and MN according to different sizes (base pairs). Bin size: 200 bp. (**C**) Comparison of the length of eccDNA (base pairs) between PN and MN. (**D**) Comparison of the distribution of partial gene–containing eccDNA that harbors 5′UTR, 3′UTR, intronic, and exonic sequences. (**E**) Schematic of the NuSeF assay. Created in BioRender. L. K. Chan (2025); https://biorender.com/9ljwwy1. (**F**) NuSeF assay comparing the DNA fibers from PN and MN. CldU and IdU were pulse labeled for 30 min each. ssDNA staining was used to visualize the integrity of DNA. (**G**) The tract length (CldU + IdU) was measured. Each dot represents a single fiber. (**H**) Left: Illustration of sorted populations. Created in BioRender. L. K. Chan (2025); https://biorender.com/bhc32os. Right: NuSeF assay showing that the DNA fiber length was decreasing from PN, large MN (MN-high), and small MN (MN-low) from HEK293T cells. (**I**) The tract length (CldU + IdU) was measured. Each dot represents a single fiber. (**J**) Cells were treated with reversine for 48 hours. The NuSeF assay was performed with treatment with/without CPT at the IdU labeling step. (**K**) The ratio of IdU to CldU was measured to study the frequency of fork stalling events under CPT treatment. (**L**) Quantitative PCR analysis of *Ifnb1* expression 16 hours after the treatment of the MN-enriched fraction on BMDMs (40 μl). Nontumor (NT) samples were treated with PBS. *n* ≥ 4. Student’s *t* test: *P < 0.05, **P < 0.01, and ****P < 0.0001 [(C), (K), and (L)]. One-way ANOVA: *P < 0.05, **P < 0.01, and ****P < 0.0001 [(A), (I), and (G)].

DNA damage in MN has been associated with chromothripsis, particularly in cells reintegrating the DNA fragments in MN back into the genome (*28*). During chromothripsis, cells acquire massive genomic rearrangements through DNA fragmentation and rejoining of DNA in a random manner (*29*). Given that we observed an increase in micronucleated hepatocytes in *Mcl1*^Δhep^ mice (Fig. 1D), we hypothesized that the presence of these structures is associated with the increase in eccDNA. To test this hypothesis, we treated AML12 cells with reversine for 48 hours to induce MN (fig. S5, A and B). Quantification of γH2AX fluorescence intensity showed that MN exhibited more severe DNA damage compared to PN (fig. S5C). By labeling the cells with 5-ethynyl-2′-deoxyuridine (EdU), we observed that some of the MN underwent active DNA replication (fig. S5D). To study MN specifically, we performed MN isolation on the basis of a method using sucrose gradient centrifugation to separate MN- and PN-rich fractions (*30*). On the basis of the DNA intensity (Hoechst 33343) and size (forward-scattered light), we sorted PN and MN from both AML12 and human embryonic kidney (HEK) 293T cells using flow cytometry and performed cirSeq (fig. S6, A and B). MN from AML12 cells showed significantly higher eccDNA levels compared to PN (Fig. 4A). Similarly to the distribution observed in livers of *Mcl1*^Δhep^ mice, the majority of eccDNA was below the length of 6000 bp (Fig. 4B). Alignment of the sequences indicated that the eccDNA also originated from all chromosomes (fig. S7A). A similar pattern was observed when comparing MN and PN from HEK293T cells (Fig. 4, A and B, and fig. S7C). The observation of MN bearing more abundant eccDNA was the same in both cell lines. Furthermore, by comparing eccDNA from MN and PN, we observed that MN tended to promote the generation of small eccDNA (Fig. 4C and fig. S7, B and D). These patterns suggested that the more abundant small eccDNA observed in *Mcl1*^Δhep^ compared to WT livers was due to higher levels of micronucleated hepatocytes. By analyzing the sequences of eccDNA identified in PN and MN, MN appeared to contain slightly more eccDNA with partial genes (fig. S7, E and F). Delving into the partial gene–containing eccDNA revealed that those with intronic sequences were more abundant in MN compared to PN (Fig. 4D).

### NuSeF assay reveals intrinsic defects in MN that exhibit stronger replication stress

To better understand how MN promote eccDNA generation, we have developed a method called nuclei-segregated DNA fiber (NuSeF) assay (Fig. 4E). This approach allowed us to distinguish DNA fibers originating from PN and MN after separation by fluorescence-activated cell sorting. Using the NuSeF assay, DNA fibers from MN of AML12 and HEK293T cells showed much shorter total tract length (CldU + IdU) (Fig. 4, F and G) compared to their corresponding PN. The reduction was observed in both the CldU and IdU tract lengths, although the IdU-to-CldU ratio was not altered (fig. S8, A to C). This suggested that MN had higher replication stress, which resulted in overall replication fork slowing. Such a pattern was consistent in both cell types. We observed an inverse correlation between the size of nuclei (PN versus MN-high versus MN-low) and the total DNA tract length (Fig. 4, H and I). Similarly to PN, MN also responded to camptothecin (CPT) treatment, showing a reduced total tract length upon induction of replication stress (fig. S8D). Treatment with CPT also resulted in a significant reduction of the IdU-to-CldU ratio in MN compared to PN (Fig. 4, J and K). This indicated that MN were more susceptible to replication stress, which might explain the observed higher DNA damage in these structures (fig. S5C). Our results suggested that although MN still retained the replication properties observed in PN, intrinsic defects in MN, such as their increased susceptibility to replication stress, could be a source of increased eccDNA generation.

### eccDNA is transferred to BMDMs indirectly through MN

To understand how eccDNA is transferred from hepatocytes to macrophages, we applied a systematic approach to study this mechanism. We hypothesized that eccDNA is released from hepatocytes into their environment. We first treated AML12 cells with reversine for 48 hours to induce MN formation. The reversine-containing medium was exchanged with a fresh medium, and the conditioned medium was collected 22 hours later. Incubation of BMDMs with the conditioned medium from AML12 cells enriched with MN for 8 or 16 hours did not result in an increased expression of *Ifnb1* (fig. S8E). Next, we tested the effect of adding linear DNA or eccDNA directly into the BMDM culture medium. BMDMs stimulated with AML12 linear gDNA (30 ng/ml) or AML12 eccDNA (30 ng/ml) also did not result in an increased expression of *Ifnb1* (fig. S8F). Notably, the DNA dosage applied here was three times higher than in the experiment with transfection (Fig. 2H). This indicates that the transfer of eccDNA to macrophages is not efficient without transfection. Given that we have shown that MN were structures enriched with eccDNA, we next wondered whether MN serve as a vehicle for delivering eccDNA to macrophages. To test this hypothesis, we modified the MN isolation protocol and obtained the MN-enriched fraction from AML12 cells treated with reversine (fig. S8G). Additional washing steps were applied on the MN-enriched fraction, and we confirmed that the purified MN-enriched fraction contained more than 91.9% of MN (fig. S8H). BMDMs treated with the MN-enriched fraction showed a significantly higher expression of *Ifnb1* compared to nontreated cells (Fig. 4L). Collectively, these results indicate that the transfer of eccDNA from hepatocytes may occur indirectly through the uptake of MN by BMDMs from the environment.

### eccDNA is increased in mouse models of liver diseases with high tumor incidence

**Fig. 5.**
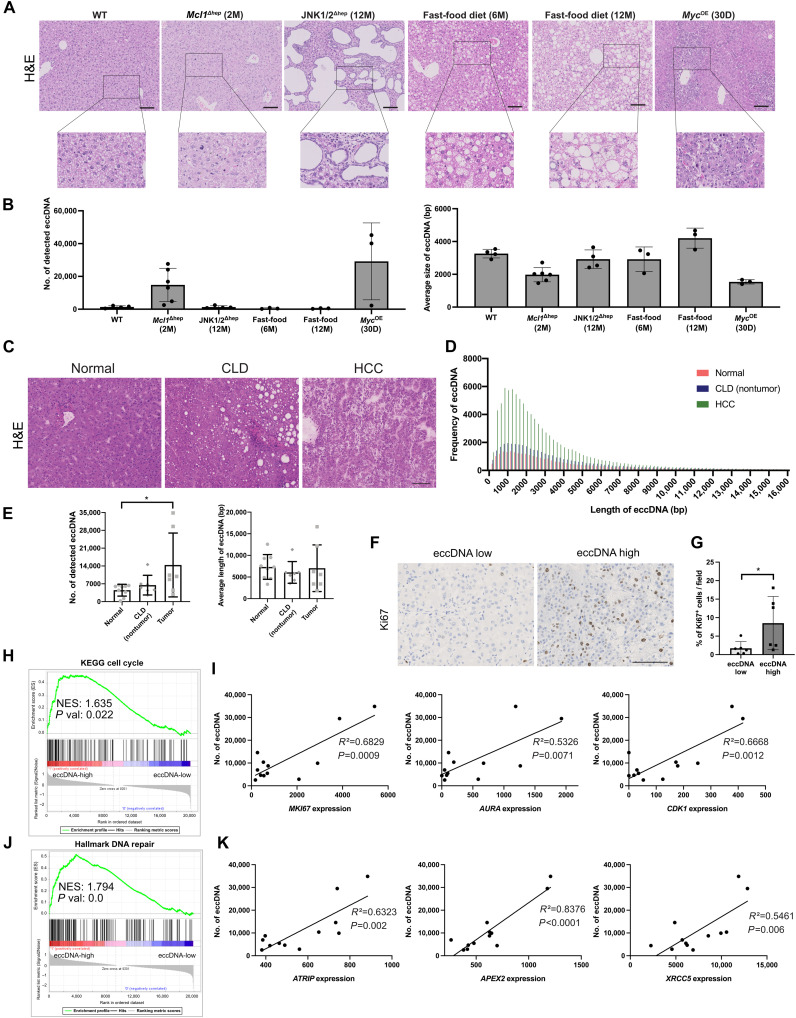
eccDNA levels are increased in mouse models with high tumor incidence and samples from patients with HCC. (**A**) Histology of mouse models of various liver diseases: 2-month-old *Mcl1*^Δhep^ liver (chronic liver injury), 12-month-old JNK1/2^Δhep^ mice (polycystic liver disease), 30-day-old LAP-tTa/tetO-c-MYC mice (MYC oncogene activation), and 6-month-old and 12-month-old fast-food diet–treated mice (fatty liver disease). Scale bars, 100 μm. H&E, hematoxylin and eosin; M, months; D, days. (**B**) CirSeq was performed from the liver tissues of these mice. The average number of detected eccDNA and their average size were compared. *n* ≥ 3. (**C**) Histology of patient tissues from normal, CLD (NT area), and HCC livers. Scale bar, 100 μm. (**D**) Plotting of frequency of eccDNA according to different sizes (base pairs). Bin size: 200 bp. *n* = 7. (**E**) Total number and average length of detected eccDNA. Each dot represents one sample. Student’s *t* test: *P < 0.05. *n* ≥ 7. (**F**) Staining of Ki67 on HCC samples from the eccDNA-high and eccDNA-low groups. Scale bar, 100 μm. (**G**) Quantification of the percentage of Ki67^+^ cells per field on the eccDNA-high and eccDNA-low CLD and HCC samples. Student’s *t* test: *P < 0.05. *n* = 6. (**H**) GSEA using RNA-seq data comparing the eccDNA-high and eccDNA-low groups showing an enriched “Cell Cycle” signature in the eccDNA-high group. (**I**) Correlation of eccDNA levels from CLD and HCC samples to their corresponding expression of proliferation-associated genes (*MKI6*, *AURA*, and *CDK1*) using normalized counts. *n* = 12. (**J**) GSEA showing an enriched “DNA Repair” signature in the eccDNA-high group. (**K**) Correlation of eccDNA levels to their corresponding expression of DNA repair–related genes (*ATRIP*, *APEX2*, and *XRCC5*) using normalized counts. *n* = 12. Pearson’s correlation coefficient and *R*^2^ were calculated in all the correlation tests.

We then aimed to find out whether the increase in eccDNA was specific to the *Mcl1*^Δhep^ mouse model. We performed cirSeq in several CLD mouse models of liver diseases, including a model of polycystic liver disease (JNK1/2^Δhep^), a model of fatty-liver disease (fast-food diet), and a model of oncogene activation (c-MYC overexpression) (Fig. 5A) (*31*–*33*). JNK1/2^Δhep^ mice at 12 months displayed multiple cystic structures in the liver. However, this phenotype was not associated with an increase in eccDNA (Fig. 5B). Mice fed with a fast-food diet developed macrovesicular steatosis but did not show any changes in eccDNA levels. In contrast, high eccDNA levels were observed in livers of *Mcl1*^Δhep^ and *Myc*^OE^ mice. These models had a reduced average size of eccDNA, suggesting that the increased eccDNA was mostly small eccDNA. Both *Mcl1*^Δhep^ and *Myc*^OE^ mouse lines had much higher tumor incidence (~50% in 12-month-old *Mcl1*^Δhep^ mice and 100% in *Myc*^OE^ mice) compared to the other models (0% in JNK1/2^Δhep^ mice and 2.5% in 12-month-old fast-food diet mice) (*31*–*33*). These data suggested a positive correlation between high eccDNA levels and the development of HCC.

### eccDNA levels are increased in human CLD and HCC tissues

To investigate whether elevated eccDNA levels could also be relevant for hepatocarcinogenesis in humans, we examined eccDNA levels in HCC and the adjacent CLD tissues in comparison to nondiseased liver tissues (Fig. 5C). CirSeq revealed that the eccDNA levels were already increased in CLD tissues and significantly elevated in HCC tissues (Fig. 5, D and E). We observed that the majority of eccDNA had a size below 8000 bp. The largest detected circular DNA in patient samples was 724,600 bp, which was still much smaller than the expected size observed in extrachromosomal DNA, a class of circular DNA frequently detectable in tumor cells (*27*). To determine whether some of these circular DNA carried complete genes, the cirSeq sequences were aligned to the human genome (GRCh38). We were able to identify eccDNA carrying at least one complete gene in all samples, with an average of 95, 62, and 73 eccDNA per sample in the normal, CLD, and HCC groups, respectively.

To analyze whether there was a correlation between the presence of full genes in eccDNA to their expression, we performed total RNA sequencing (RNA-seq). A comparison of samples that contained full genes present in eccDNA with the rest of the samples did not reveal a significant up-regulation of these genes, suggesting that these genes were most likely not amplified in the circular form (fig. S9, A to C). Given that eccDNA levels were increased in CLD and HCC tissue samples, we divided these samples into eccDNA-high and eccDNA-low groups on the basis of their eccDNA levels. We observed that the percentage of Ki67^+^ cells in the eccDNA-high group was significantly higher than in the eccDNA-low group (Fig. 5, F and G). eccDNA-high samples also showed a tendency toward increased infiltration by CD11b^+^ cells (fig. S10A). We then performed gene set enrichment analysis (GSEA) and observed a significant enrichment of the “Cell Cycle” signature in the eccDNA-high group (Fig. 5H). Correlation analyses revealed that the level of eccDNA was strongly correlated with the expression of proliferation-associated genes, e.g., *MKI67* (gene encoding Ki67), *AURKA* (gene encoding aurora kinase A), and *CDK1* (gene encoding CDK1) (Fig. 5I). In line, a statistically significant positive correlation was found between the eccDNA levels and the percentage of Ki67^+^ cells (fig. S10B). Furthermore, we observed an enrichment of the “Hallmark DNA Repair” signature in the eccDNA-high group (Fig. 5J). A strong positive correlation was also observed between eccDNA levels and the expression of various genes in several DNA repair mechanisms: *ATRIP*, which is essential for recruiting ATR (ataxia telangiectasia and Rad3-related protein) in response to DNA double-strand break (DSB) as part of the DNA damage response; *APEX2*, which encodes an apurinic/apyrimidinic endodeoxyribonuclease that functions in the DNA base excision repair; and XRCC5, which encodes Ku80 that, together with Ku70, binds to DSB and promotes nonhomologous end-joining (Fig. 5K and fig. S10C). Collectively, these results suggested that patient samples with strong proliferation and consequently increased replication stress and DNA damage tended to generate higher levels of eccDNA.

### Deletion of *Sting1* reduces inflammation and tumor incidence in *Mcl1*^Δhep^ mice

**Fig. 6.**
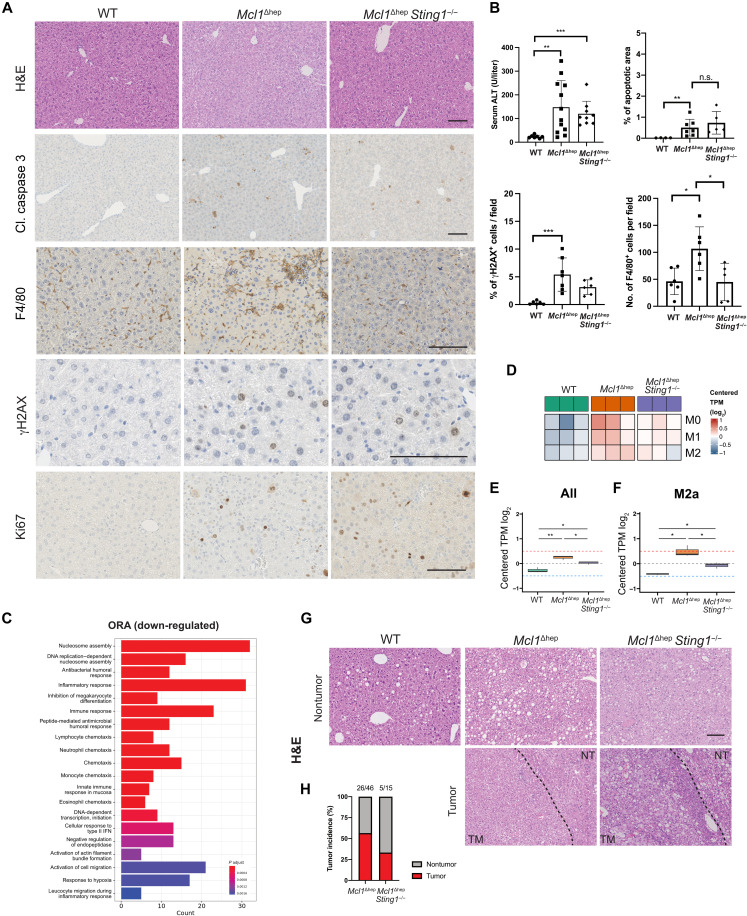
STING knockout reduces immune cell infiltration and tumor incidence in *Mcl1*^Δhep^ mice. (**A**) Histological analyses of 2-month-old mice showed no differences in apoptosis (cleaved caspase 3), DNA damage (γH2AX), and proliferation (Ki67) but a reduction in macrophage infiltration (F4/80) in *Mcl1*^Δhep^ *Sting1*^−/−^ mice. Scale bar, 100 μm. (**B**) Measurement of serum alanine transaminase (ALT) levels from blood samples collected at the 2-month time point. Quantification of the cleaved caspase 3 staining as a percentage of total area. Quantification of the number of γH2AX^+^ hepatocytes per field. Quantification of the number of F4/80^+^ cells per field. Student’s *t* test: *P < 0.05, **P < 0.01, and ***P < 0.001. n.s., not significant. (**C**) *Mcl1*^Δhep^ *Sting1*^−/−^ mice showed a significant down-regulation of gene signatures involved in immune responses and immune cell recruitment. ORA, overrepresentation analysis. (**D**) Heatmap showing averaged M0-, M1-, and M2-associated genes. (**E** and **F**) Box plot comparing the log_2_-centered TPM of the averaged M0-, M1-, and M2-associated genes together or M2a-associated genes. One-way ANOVA followed by post hoc pairwise two-sided Student’s *t* test: *P < 0.05 and **P < 0.01. (**G**) Histology of 12-month livers showing the tumor (TM) area and nontumor (NT) area. Scale bar, 100 μm. (**H**) Tumor incidence showing the percentage of mice in the corresponding genotypes that displayed at least one tumor in the liver.

Given that the effect of eccDNA on immune cells in inducing *Ifnb1* and *Tnfa* expression was dependent on the cGAS-STING pathway (Fig. 2H and fig. S3I), we sought to determine whether the phenotype of *Mcl1*^Δhep^ mice would be affected by blocking this pathway. To this aim, we crossed *Sting1*^−/−^ mice with *Mcl1*^Δhep^ mice to generate an *Mcl1*^Δhep^ *Sting1*^−/−^ mouse line. We confirmed the knockout status of the *Sting1*^−/−^ mice by Western blotting and observed that splenocytes from these mice were not able to induce IRF3 phosphorylation after DMXAA (5,6-dimethylxanthenone-4-acetic acid; a murine STING1 agonist) stimulation (fig. S11A). *Mcl1*^Δhep^ *Sting1*^−/−^ mice showed no significant difference in liver damage [serum alanine transaminase (ALT)], level of apoptosis (cl. caspase 3), proliferation (Ki67), or DNA damage (γH2AX) when compared to *Mcl1*^Δhep^ mice (Fig. 6, A and B). Given the expression pattern of STING in the liver (fig. S2C), the effect of inhibiting the STING pathway was assumed to affect immune cells. To test this, total RNA-seq and gene signature analyses were performed on *Mcl1*^Δhep^ *Sting1*^−/−^ liver samples using *Mcl1*^Δhep^ mice as a control. Overrepresentation analysis (ORA) indicated that most of down-regulated signatures in the *Mcl1*^Δhep^ *Sting1*^−/−^ liver were related to immune response (Fig. 6C). Various ISGs were found to be significantly down-regulated upon *Sting1* deletion (fig. S11B). Using GSEA, we observed a depletion of signatures involved in immune cell chemotaxis in *Mcl1*^Δhep^ *Sting1*^−/−^ mice, e.g., macrophage migration and granulocyte chemotaxis (fig. S11C). In line, quantification of F4/80-positive cells showed that STING knockout reduced the number of macrophages/Kuffer cells in the *Mcl1*^Δhep^ liver (Fig. 6B). Such a reduction could be associated with a down-regulation of key chemokines of macrophages, e.g., CXCL10, in *Mcl1*^Δhep^ *Sting1*^−/−^ mice (fig. S11B).

Given that the polarization of macrophages into the M1 or M2 phenotype can have a distinct effect on the microenvironment and remarkably influence liver tumorigenesis, we investigated the different macrophage subpopulations in the WT, *Mcl1*^Δhep^, and *Mcl1*^Δhep^ *Sting1*^−/−^ mice. We extracted the expression levels of gene clusters associated with M0, M1, and M2 macrophages and observed that all three subpopulations were elevated in the *Mcl1*^Δhep^ group compared to WT (Fig. 6, D and E, and fig. S11, D and E). When comparing the *Mcl1*^Δhep^ mice to *Mcl1*^Δhep^ *Sting1*^−/−^ mice, we observed a significant reduction in the latter when analyzing all these macrophage-associated genes together (Fig. 6E). However, when looking at individual M0, M1, or M2 gene clusters, only a strong tendency of reduced expression was found in the *Mcl1*^Δhep^ *Sting1*^−/−^ group (fig. S11E). M2 macrophages often exhibit an immunosuppressive function in the tumor microenvironment and can be further classified as M2a, M2b, M2c, and M2d subsets (*34*). The M2a subset has been shown to exhibit a protumoral activity (*35*). By analyzing a gene cluster associated with the M2a macrophages, we observed that this subset was significantly elevated in the *Mcl1*^Δhep^ mice compared to WT mice. However, STING deletion led to a significant reduction of the M2a subset in the *Mcl1*^Δhep^ mice (Fig. 6F). Given that we observed a reduced immune response and subsets of protumorigenic macrophages upon *Sting1* deletion, we compared cohorts of these mice at the age of 12 months. *Mcl1*^Δhep^ *Sting1*^−/−^ mice showed a reduced tumor incidence of 33.33% compared to 56.52% in the *Mcl1*^Δhep^ group (Fig. 6, G and H). These results suggested that the deletion of STING reduced the immune response and that such a disrupted inflammatory microenvironment led to a reduction in the tumor incidence, highlighting the importance of the cGAS-STING pathway in eccDNA-mediated inflammation in *Mcl1*^Δhep^ livers.

## DISCUSSION

Two decades have passed since the presence of MN in human CLD and HCC tissues was first described (*36*). However, it is still not fully understood how the presence of MN is linked to HCC carcinogenesis. The results of this study shed light on this old observation and suggest a close connection between the formation of eccDNA, the immune response, and the development of liver cancer. MN are known to arise from chromosome missegregation. However, in cells experiencing replication stress, MN can also contain chromatin fragments caused by DNA damage (*12*). Using *Mcl1*^Δhep^ mice as a CLD model, DNA fiber assay indicated increased replication stress in MCL1-deficient hepatocytes. Prolonged replication fork stalling often causes fork collapses, leading to DSB (*37*). The high replication stress caused by apoptosis-triggered hyperproliferation in MCL1-deficient hepatocytes can in turn lead to higher DNA damage and MN, which ultimately promote apoptosis in a positive feedback loop. Although apoptosis has been shown to promote the formation of eccDNA, its biogenesis has also been shown to be associated with other stress conditions or DNA damage (*20*, *21*). Our data suggest that the formation and accumulation of eccDNA occur before apoptosis through the formation of MN.

To gain insight into the relationship between MN and eccDNA formation, we aimed at developing a method allowing us to study replication stress directly in MN. This approach, designated the NuSeF assay, enabled us to distinguish DNA fibers originating from MN and PN, in contrast to the conventional protocol that examines bulk DNA fibers. The NuSeF assay revealed that MN were more susceptible to replication stress, as indicated by replication fork slowing. This observation confirms the overall higher DNA damage levels in MN compared to PN detected by imaging methods. Using cirSeq, eccDNA was detected in both PN and MN. The fact that eccDNA was detected in PN may explain the previous report that eccDNA was found in various healthy tissues (*19*). Presumably, the main source of eccDNA from healthy tissue is from PN, as healthy cells generally do not contain MN. Even though MN contained more abundant eccDNA, as shown in this study, their eccDNA is much smaller. This may partly reflect the more abundant but smaller eccDNA observed in *Mcl1*^Δhep^ livers because of the increased proportion of micronucleated cells. The micronucleated hepatocytes in *Mcl1*^Δhep^ livers and the MN-containing AML12 cells after reversine treatment were nonapoptotic. A potential limitation of our study is that we could not completely exclude a contribution of apoptosis to eccDNA biogenesis in the *Mcl1*^Δhep^ model, as apoptosis was one of the effects of MCL1 deletion in hepatocytes. Given that excessive DNA damage may ultimately promote apoptosis, it is more likely that eccDNA is generated after MN formation and the generation is later enhanced by apoptosis. This is in line with the current understanding that eccDNA can be produced by multiple mechanisms (*38*). Further application of the NuSeF assay, in combination with cirSeq, on cells exposed to various genotoxic insults or in experimental designs to restore replication defects within MN will contribute to a better understanding of the MN biology and possibly reveal further mechanisms of eccDNA generation.

The circular nature of eccDNA is likely to provide additional stability and therefore increase the potency in inducing the immune response (*20*). We observed that even at low dosage, eccDNA can induce *Ifnb1* expression in BMDMs. The potency can also be an effect of eccDNA resistance to intracellular exonuclease such as TREX1 (three prime repair exonuclease 1), which has an inhibitory function for the activation of the cGAS-STING pathway (*39*). However, it is still unclear whether eccDNA is actively secreted by damaged hepatocytes or whether they are released during cell death. The ability of eccDNA to induce a strong response in immune cells suggests that they are acting as damage-associated molecular pattern molecules. Furthermore, it is also unclear whether eccDNA is secreted locally to activated adjacent immune cells or whether they have a systemic effect when present in the circulation as part of the circulating DNA. Cell-free eccDNA has been reported in the plasma of both mice and humans, which suggests a possibility to reach distant targets (*40*). In this study, we demonstrate an alternative mechanism that facilitates the transfer of eccDNA to macrophages. Neither the addition of eccDNA directly to the culture medium nor the application of conditioned medium derived from reversine-treated hepatocytes was able to stimulate *Ifnb1* expression in BMDMs. In contrast, treatment of BMDMs with the MN-enriched fraction strongly induced *Ifnb1* expression in BMDMs. Given that MN are structures enriched with eccDNA, they could serve as vehicles for delivering eccDNA to macrophages. Further studies are needed to investigate whether hepatocytes actively release MN into the environment.

Although an increase in DNA damage may eventually increase the number of accumulated mutations, which ultimately can drive neoplastic transformation, we observed an essential role of the immune cells in the carcinogenesis process in the *Mcl1*^Δhep^ model. Our results suggest a cross-talk between hepatocytes and the microenvironment, with eccDNA serving as a link connecting the increased DNA damage in hepatocytes to the activation of the cGAS-STING pathway in the immune cells. This connection is essential because hepatocytes do not express STING (*14*, *16*). Inhibition of this pathway reduced the overall immune cell infiltration in *Mcl1*^Δhep^ livers and reduced the tumor incidence at 12 months. Inflammation has long been known to be associated with HCC development (*41*). Therefore, our results suggest that carcinogenesis in *Mcl1*^Δhep^ mice, which very faithfully recapitulate the CLD pathophysiology, is mediated through a non–cell-autonomous mechanism.

In the present study, we showed that the effect of eccDNA on immune cells is dependent on both cGAS and STING. In animal models receiving high-fat diet or methionine- and choline-deficient diet, constitutive knockout of STING and selective knockout of STING in myeloid cells both led to a reduced nonalcoholic steatohepatitis phenotype, which includes attenuated liver steatosis, inflammation, and fibrosis (*14*, *16*). These studies also highlighted the observation of a lack of STING expression in hepatocytes, as confirmed in the current study. Such an observation also supports the concept that hepatocytes are susceptible to hepatitis B virus infection because of a lack of the DNA sensing pathway and their inability to induce a type 1 interferon response after viral infection (*15*). Therefore, the postulated role of STING in eliciting a senescence-associated secretory phenotype might be important in other cell types but may not be relevant in hepatocytes (*42*). Because of the different spatial expressions of cGAS and STING, selective inhibition of these components may result in different outcomes. Several studies reported a noncanonical role of cGAS in regulating senescence, cell death, and DNA repair (*43*–*46*). Similarly, noncanonical activation of STING after DNA damage has been reported through the recruitment of ATM (ataxia-telangiectasia mutated) and IFI16 (interferon-γ–inducible protein 16), leading to the activation of the downstream NF-κB signaling (*47*). The relevance of these noncanonical functions in hepatocytes, especially in the pathogenesis of liver diseases, still requires further elucidation.

In summary, this study provides evidence that eccDNA, generated by an accumulation of MN, acts as a link in a cross-talk between hepatocytes and immune cells to maintain the immune response in the liver. Our results show that carcinogenesis in *Mcl1*^Δhep^ mice is mediated by a non–cell-autonomous mechanism, with the cross-talk between hepatocytes and immune cells playing a pivotal role. The NuSeF assay is a previously unknown approach to enable the study of replication fork dynamics in MN, where here led to the discovery of higher replication stress in MN.

## MATERIALS AND METHODS

### Experimental design

Although the increase in DNA damage and genomic instability in hepatocytes may be directly linked to carcinogenesis in CLD, it is still unclear whether the carcinogenesis from CLD to HCC is mediated by a cell-autonomous or non–cell-autonomous mechanism. Here, we used several murine liver disease models and human CLD and HCC tissues, in combination with cultured cells, to study the relationship between MN and eccDNA in liver inflammation and HCC development. Previously unindentified mechanisms involving interaction between hepatocytes and immune cells in the liver were uncovered, suggesting a non–cell-autonomous mechanism in the maintenance of chronic inflammation and hepatocarcinogenesis.

### Mouse lines and patient tissue

*Mcl1*^Δhep^ mice were generated by crossing Alb-Cre transgenic mice with *Mcl1* floxed mice, as described previously (*8*). Livers of JNK1/2^Δhep^ mice, mice treated for 6 or 12 months of fast-food diet, and *Myc*^OE^ mice were obtained as described previously (*31*–*33*). *Sting1*^−/−^ mice were provided by T. Buch (Laboratory Animal Services Center, Universität Zürich). *Mcl1*^Δhep^ *Sting1*^−/−^ mice were generated by crossing *Mcl1*^Δhep^ mice with *Sting1*^−/−^ mice. *Cgas*^−/−^ mice were purchased from JAX. WT, *Mcl1*^Δhep^, and *Mcl1*^Δhep^ *Sting1*^−/−^ mice were analyzed at the ages of 2 and 12 months. Experiments were performed using animals of both sexes. Snap-frozen or formalin-fixed, paraffin-embedded human liver samples were provided by the biobank of the Department of Pathology and Molecular Pathology, University Hospital Zurich, for morphological and molecular analyses.

### NuSeF assay

Cells were pulse labeled with two thymidine analogs, CldU and IdU, for 30 min each. Labeled cells were harvested and treated with cytochalasin B for 30 min on ice. PN and MN were enriched in fractions by sucrose gradient centrifugation. Fluorescence-activated cell sorting was performed on these enriched fractions to obtain pure PN and MN. Purified nuclei were lysed, and the DNA content was spread on glass slides. The slides were then incubated with anti-CldU (BD Bioscience, no. 347580) and anti-IdU (Abcam, no. ab6326) primary antibodies, followed by secondary antibodies. Single-stranded DNA (ssDNA) (DSHB TROMA-III) counterstaining was performed to visualize the integrity of DNA fibers. The fiber tract length was quantified and compared. A detailed NuSeF assay protocol is available in the Supplementary Text and has been uploaded to www.protocols.io (DOI: dx.doi.org/10.17504/protocols.io.bp2l6znpkgqe/v1).

### Primary hepatocyte and immune cell isolation from the liver

Hepatocyte isolation was performed according to the proposed protocol (*48*). Briefly, mice were first anesthetized with ketamine (100 mg/kg) and xylazine (16 mg/kg). The liver was first perfused with the preperfusion buffer (0.5 mM EDTA/20 mM Hepes/Hanks’ balanced salt solution) for 5 min and then switched to a perfusion solution [20 mM Hepes/1× penicillin/streptomycin/3 mM CaCl_2_/Dulbecco’s modified Eagle’s medium/F12/Liberase (0.2 mg/ml; Roche, no. 05401127001)] for 10 min. The liver was then transferred to a petri dish with 10 ml of Wash solution (4% FBS/1× penicillin/streptomycin/Willian’s E medium), and hepatocytes were gently released from the liver using forceps. The Wash solution containing cells was filtered through a 70-μm cell strainer (Falcon, no. 352350) and stored on ice. The cell suspension was centrifuged at 20*g* for 3 min. The pellet was used for hepatocyte isolation, and the supernatant, which contained NPCs, was transferred to another 50-ml Falcon tube for immune cell isolation. For hepatocyte purification, the pellet was resuspended with 10 ml of Wash solution, mixed with 10 ml of 90% Percoll solution (Cytiva, no. GE-17-0891-01), and centrifuged at 200*g* for 10 min. This step was repeated one more time to increase the purity of viable cells. The pellet was then resuspended in the Wash solution and seeded at a density of 3 × 10^5^ cells per well in a collagen-precoated 24-well plate. For isolating immune cells, the volume was brought to 50 ml using the Wash solution. The solution was centrifuged at 20*g* for 3 min, and the supernatant was transferred to another 50-ml Falcon tube. This step was repeated for additional two times. The transferred supernatant was then centrifuged at 2000 rpm for 5 min. The immune cell pellet was resuspended in 10 ml of 36% Percoll solution and centrifuged at 2000 rpm for 20 min at 4°C. Red blood cells were lysed with 1× RBC lysis buffer (G Biosciences, no. 786-649) at room temperature for 5 min. The immune cells were washed once with 10 ml of phosphate-buffered saline (PBS) and pelleted followed by snap-freezing and storage. The purity of the isolated cells was examined using Western blotting for hepatocyte- and immune cell–specific markers (albumin and CD45, respectively).

### DNA fiber assay

For the DNA fiber assay on primary hepatocytes, primary hepatocytes were isolated as described above. Hepatocytes were seeded in collagen-coated six-well plates at a density of 20% after isolation. Hepatocytes were cultured for 24 hours before the assay. On the day of the experiment, the hepatocyte medium was removed from the wells and first replaced with 19 mM CldU in a fresh medium followed by exchanging the medium with 28.24 mM IdU in a fresh medium at 30-min intervals. The wells were washed twice with prewarmed PBS in between CldU- and IdU-containing media. After the IdU incubation, cells were washed twice with cold PBS and trypsinized. Cells were harvested and stored on ice until DNA spread. Approximately 1000 cells in 3 μl were transferred onto a glass slide and then mixed with 7 μl of lysis buffer (200 mM tris-HCl, pH 7.4/50 mM EDTA/0.5% SDS). After 5 min of lysis at room temperature, the slides were tilted at an angle of 45° to promote the spreading of DNA by gravity. The slides were then dried and fixed with methanol/acetic acid (3:1) for 20 min at 4°C. Slides were rehydrated in PBS and then denatured with 2.5 M HCl for 1 hour at room temperature. These slides were then washed five times with PBS and blocked with blocking buffer (2% bovine serum albumin/0.1% Tween 20/PBS) for 1 hour at room temperature. The slides were then incubated with anti-CldU (BD Bioscience, no. 347580) and anti-IdU (Abcam, no. ab6326) primary antibodies overnight at 4°C, followed by 2 hours of secondary antibodies at room temperature. ssDNA (DSHB TROMA-III) counterstaining was performed to visualize the integrity of DNA fibers. The stained slides were mounted with ProLong Gold Antifade mounting medium (Invitrogen, no. P36930). DNA fiber images were acquired using a Leica DM6 microscope.

### Study approval

All animal experiments were approved by the Zurich Cantonal Veterinary Office (licenses: ZH193/2020 and ZH104/2019). This study was approved by the internal review board of the University Hospital Zurich and the Cantonal Ethics Committee of Zurich, Switzerland (KEK-ZH no. 2013-0382). Where required by local regulations governing the use of human tissue samples, informed consent was obtained in all cases.

### Statistical analysis

Statistical analyses were performed as indicated in the figure legends. For a comparison between the two groups, Student’s *t* test was used. For a comparison of more than two groups, a one-way analysis of variance (ANOVA) was used. For analyzing the cell type gene set, the mean-centered log_2_ transcript per million reads (TPM) gene expression was calculated per sample. A global one-way ANOVA was performed, followed by post hoc pairwise two-sided Student’s *t* tests. For all analyses, *P < 0.05, **P < 0.01, ***P < 0.001, and ****P < 0.0001. Sample numbers (*n*) are indicated in figure legends. All graphs show the means ± SD. Each dot in all dot plots indicates each sample or individual quantified unit. Detailed information is provided in the corresponding figure legend. Statistical tests were performed using GraphPad Prism 10 or R. Extended technical descriptions are available in the Supplementary Text.

## Supplementary Materials

### This PDF file includes:

Supplementary Text Figs. S1 to S11 Tables S1 to S7 References

## References

[1] H. Rumgay, M. Arnold, J. Ferlay, O. Lesi, C. J. Cabasag, J. Vignat, M. Laversanne, K. A. McGlynn, I. Soerjomataram, Global burden of primary liver cancer in 2020 and predictions to 2040. J. Hepatol. 77, 1598–1606 (2022). 36208844 10.1016/j.jhep.2022.08.021 PMC9670241

[2] J. D. Yang, P. Hainaut, G. J. Gores, A. Amadou, A. Plymoth, L. R. Roberts, A global view of hepatocellular carcinoma: Trends, risk, prevention and management. Nat. Rev. Gastroenterol. Hepatol. 16, 589–604 (2019). 31439937 10.1038/s41575-019-0186-y PMC6813818

[3] P. Le, M. Tatar, S. Dasarathy, N. Alkhouri, W. H. Herman, G. B. Taksler, A. Deshpande, W. Ye, O. A. Adekunle, A. M. Cullough, M. B. Rothberg, Estimated burden of metabolic dysfunction-associated steatotic liver disease in US adults, 2020 to 2050. JAMA Netw. Open 8, e2454707 (2025). 39821400 10.1001/jamanetworkopen.2024.54707 PMC11742522

[4] G. Feng, G. Targher, C. D. Byrne, Y. Yilmaz, V. Wai-Sun Wong, C. R. Adithya Lesmana, L. A. Adams, J. Boursier, G. Papatheodoridis, M. el-Kassas, N. Méndez-Sánchez, S. Sookoian, L. Castera, W. K. Chan, F. Ye, S. Treeprasertsuk, H. Cortez-Pinto, H. H. Yu, W. Kim, M. Romero-Gómez, A. Nakajima, K. M. Win, S. U. Kim, A. G. Holleboom, G. Sebastiani, P. Ocama, J. D. Ryan, M. Lupșor-Platon, H. Ghazinyan, M. al-Mahtab, S. Hamid, N. Perera, K. A. Alswat, Q. Pan, M. T. Long, V. Isakov, M. Mi, M. Arrese, A. J. Sanyal, S. K. Sarin, N. C. Leite, L. Valenti, P. N. Newsome, H. Hagström, S. Petta, H. Yki-Järvinen, J. M. Schattenberg, M. I. Castellanos Fernández, I. A. Leclercq, G. Aghayeva, A. N. Elzouki, A. Tumi, A. I. Sharara, A. Labidi, F. M. Sanai, K. Matar, M. al-Mattooq, M. W. Akroush, M. Benazzouz, N. Debzi, M. Alkhatry, S. Barakat, S. A. al-Busafi, J. Rwegasha, W. Yang, A. Adwoa, C. K. Opio, M. Sotoudeheian, Y. J. Wong, J. George, M. H. Zheng, Global burden of metabolic dysfunction-associated steatotic liver disease, 2010 to 2021. JHEP Rep. 7, 101271 (2025). 39980749 10.1016/j.jhepr.2024.101271 PMC11840544

[5] M. Ringelhan, D. Pfister, T. O’Connor, E. Pikarsky, M. Heikenwalder, The immunology of hepatocellular carcinoma. Nat. Immunol. 19, 222–232 (2018). 29379119 10.1038/s41590-018-0044-z

[6] A. Weber, R. Boger, B. Vick, T. Urbanik, J. Haybaeck, S. Zoller, A. Teufel, P. H. Krammer, J. T. Opferman, P. R. Galle, M. Schuchmann, M. Heikenwalder, H. Schulze-Bergkamen, Hepatocyte-specific deletion of the antiapoptotic protein myeloid cell leukemia-1 triggers proliferation and hepatocarcinogenesis in mice. Hepatology 51, 1226–1236 (2010). 20099303 10.1002/hep.23479 PMC2936921

[7] H. Nakagawa, A. Umemura, K. Taniguchi, J. Font-Burgada, D. Dhar, H. Ogata, Z. Zhong, M. A. Valasek, E. Seki, J. Hidalgo, K. Koike, R. J. Kaufman, M. Karin, ER stress cooperates with hypernutrition to trigger TNF-dependent spontaneous HCC development. Cancer Cell 26, 331–343 (2014). 25132496 10.1016/j.ccr.2014.07.001 PMC4165611

[8] Y. Boege, M. Malehmir, M. E. Healy, K. Bettermann, A. Lorentzen, M. Vucur, A. K. Ahuja, F. Böhm, J. C. Mertens, Y. Shimizu, L. Frick, C. Remouchamps, K. Mutreja, T. Kähne, D. Sundaravinayagam, M. J. Wolf, H. Rehrauer, C. Koppe, T. Speicher, S. Padrissa-Altés, R. Maire, J. M. Schattenberg, J. S. Jeong, L. Liu, S. Zwirner, R. Boger, N. Hüser, R. J. Davis, B. Müllhaupt, H. Moch, H. Schulze-Bergkamen, P. A. Clavien, S. Werner, L. Borsig, S. A. Luther, P. J. Jost, R. Weinlich, K. Unger, A. Behrens, L. Hillert, C. Dillon, M. di Virgilio, D. Wallach, E. Dejardin, L. Zender, M. Naumann, H. Walczak, D. R. Green, M. Lopes, I. Lavrik, T. Luedde, M. Heikenwalder, A. Weber, A dual role of caspase-8 in triggering and sensing proliferation-associated DNA damage, a key determinant of liver cancer development. Cancer Cell 32, 342–359.e10 (2017). 28898696 10.1016/j.ccell.2017.08.010 PMC5598544

[9] L. W. Thomas, C. Lam, S. W. Edwards, Mcl-1; The molecular regulation of protein function. FEBS Lett. 584, 2981–2989 (2010). 20540941 10.1016/j.febslet.2010.05.061

[10] D. A. Jackson, A. Pombo, Replicon clusters are stable units of chromosome structure: Evidence that nuclear organization contributes to the efficient activation and propagation of s phase in human cells. J. Cell Biol. 140, 1285–1295 (1998). 9508763 10.1083/jcb.140.6.1285 PMC2132671

[11] C. J. Merrick, D. Jackson, J. F. X. Diffley, Visualization of altered replication dynamics after DNA damage in human cells. J. Biol. Chem. 279, 20067–20075 (2004). 14982920 10.1074/jbc.M400022200

[12] M. Di Bona, S. F. Bakhoum, Micronuclei and cancer. Cancer Discov. 14, 214–226 (2024). 38197599 10.1158/2159-8290.CD-23-1073 PMC11265298

[13] J. Kwon, S. F. Bakhoum, The cytosolic DNA-sensing cGAS-STING pathway in cancer. Cancer Discov. 10, 26–39 (2020). 31852718 10.1158/2159-8290.CD-19-0761 PMC7151642

[14] X. Luo, H. Li, L. Ma, J. Zhou, X. Guo, S. L. Woo, Y. Pei, L. R. Knight, M. Deveau, Y. Chen, X. Qian, X. Xiao, Q. Li, X. Chen, Y. Huo, K. McDaniel, H. Francis, S. Glaser, F. Meng, G. Alpini, C. Wu, Expression of STING is increased in liver tissues from patients with NAFLD and promotes macrophage-mediated hepatic inflammation and fibrosis in mice. Gastroenterology 155, 1971–1984.e4 (2018). 30213555 10.1053/j.gastro.2018.09.010 PMC6279491

[15] M. K. Thomsen, R. Nandakumar, D. Stadler, A. Malo, R. M. Valls, F. Wang, L. S. Reinert, F. Dagnæs-Hansen, A. K. Hollensen, J. G. Mikkelsen, U. Protzer, S. R. Paludan, Lack of immunological DNA sensing in hepatocytes facilitates hepatitis B virus infection. Hepatology 64, 746–759 (2016). 27312012 10.1002/hep.28685

[16] Y. Yu, Y. Liu, W. An, J. Song, Y. Zhang, X. Zhao, STING-mediated inflammation in Kupffer cells contributes to progression of nonalcoholic steatohepatitis. J. Clin. Invest. 129, 546–555 (2019). 30561388 10.1172/JCI121842 PMC6355218

[17] A. Ablasser, M. Goldeck, T. Cavlar, T. Deimling, G. Witte, I. Röhl, K. P. Hopfner, J. Ludwig, V. Hornung, cGAS produces a 2′-5′-linked cyclic dinucleotide second messenger that activates STING. Nature 498, 380–384 (2013). 23722158 10.1038/nature12306 PMC4143541

[18] A. Ablasser, J. L. Schmid-Burgk, I. Hemmerling, G. L. Horvath, T. Schmidt, E. Latz, V. Hornung, Cell intrinsic immunity spreads to bystander cells via the intercellular transfer of cGAMP. Nature 503, 530–534 (2013). 24077100 10.1038/nature12640 PMC4142317

[19] H. D. Moller, M. Mohiyuddin, I. Prada-Luengo, M. Reza Sailani, J. F. Halling, P. Plomgaard, L. Maretty, A. J. Hansen, M. P. Snyder, H. Pilegaard, H. Y. K. Lam, B. Regenberg, Circular DNA elements of chromosomal origin are common in healthy human somatic tissue. Nat. Commun. 9, 1069 (2018). 29540679 10.1038/s41467-018-03369-8 PMC5852086

[20] Y. Wang, M. Wang, M. N. Djekidel, H. Chen, D. Liu, F. W. Alt, Y. Zhang, eccDNAs are apoptotic products with high innate immunostimulatory activity. Nature 599, 308–314 (2021). 34671165 10.1038/s41586-021-04009-w PMC9295135

[21] L. W. Dillon, P. Kumar, Y. Shibata, Y.-H. Wang, S. Willcox, J. D. Griffith, Y. Pommier, S. Takeda, A. Dutta, Production of extrachromosomal MicroDNAs is linked to mismatch repair pathways and transcriptional activity. Cell Rep. 11, 1749–1759 (2015). 26051933 10.1016/j.celrep.2015.05.020 PMC4481157

[22] Y. Wang, M. Wang, Y. Zhang, Purification, full-length sequencing and genomic origin mapping of eccDNA. Nat. Protoc. 18, 683–699 (2023). 36517607 10.1038/s41596-022-00783-7

[23] G. Sankar, M. Karin, Missing pieces in the NF-κB puzzle. Cell 109, S81–S96 (2002). 11983155 10.1016/s0092-8674(02)00703-1

[24] S. Luecke, A. Holleufer, M. H. Christensen, K. L. Jønsson, G. A. Boni, L. K. Sørensen, M. Johannsen, M. R. Jakobsen, R. Hartmann, S. R. Paludan, cGAS is activated by DNA in a length-dependent manner. EMBO Rep. 18, 1707–1715 (2017). 28801534 10.15252/embr.201744017 PMC5623850

[25] Y. Liu, X. Chen, Y. Zhao, X. Y. Wang, Y. W. Luo, L. Chen, W. Wang, S. Zhong, M. Hu, Z. Dai, J. Jiang, X. Wang, H. Ji, X. X. Cheng, A. Zheng, J. Zuo, H. Liu, D. Ma, Z. Luo, F. Cao, S. Hu, A. L. Huang, K. F. Tang, Small cytosolic double-stranded DNA represses cyclic GMP-AMP synthase activation and induces autophagy. Cell Rep. 42, 112852 (2023). 37481718 10.1016/j.celrep.2023.112852

[26] A. Henssen, I. MacArthur, R. Koche, H. Dorado-García, A. Henssen, Purification and sequencing of large circular DNA from human cells. Protoc. Exch., 10.1038/protex.2019.006 (2019).

[27] R. P. Koche, E. Rodriguez-Fos, K. Helmsauer, M. Burkert, I. C. MacArthur, J. Maag, R. Chamorro, N. Munoz-Perez, M. Puiggròs, H. Dorado Garcia, Y. Bei, C. Röefzaad, V. Bardinet, A. Szymansky, A. Winkler, T. Thole, N. Timme, K. Kasack, S. Fuchs, F. Klironomos, N. Thiessen, E. Blanc, K. Schmelz, A. Künkele, P. Hundsdörfer, C. Rosswog, J. Theissen, D. Beule, H. Deubzer, S. Sauer, J. Toedling, M. Fischer, F. Hertwig, R. F. Schwarz, A. Eggert, D. Torrents, J. H. Schulte, A. G. Henssen, Extrachromosomal circular DNA drives oncogenic genome remodeling in neuroblastoma. Nat. Genet. 52, 29–34 (2020). 31844324 10.1038/s41588-019-0547-z PMC7008131

[28] C. Z. Zhang, A. Spektor, H. Cornils, J. M. Francis, E. K. Jackson, S. Liu, M. Meyerson, D. Pellman, Chromothripsis from DNA damage in micronuclei. Nature 522, 179–184 (2015). 26017310 10.1038/nature14493 PMC4742237

[29] P. J. Stephens, C. D. Greenman, B. Fu, F. Yang, G. R. Bignell, L. J. Mudie, E. D. Pleasance, K. W. Lau, D. Beare, L. A. Stebbings, S. McLaren, M. L. Lin, D. McBride, I. Varela, S. Nik-Zainal, C. Leroy, M. Jia, A. Menzies, A. P. Butler, J. W. Teague, M. A. Quail, J. Burton, H. Swerdlow, N. P. Carter, L. A. Morsberger, C. Iacobuzio-Donahue, G. A. Follows, A. R. Green, A. M. Flanagan, M. R. Stratton, P. A. Futreal, P. J. Campbell, Massive genomic rearrangement acquired in a single catastrophic event during cancer development. Cell 144, 27–40 (2011). 21215367 10.1016/j.cell.2010.11.055 PMC3065307

[30] E. Toufektchan, J. Maciejowski, Purification of micronuclei from cultured cells by flow cytometry. STAR Protoc. 2, 100378 (2021). 33778777 10.1016/j.xpro.2021.100378 PMC7982749

[31] K. Müller, H. Honcharova-Biletska, C. Koppe, M. Egger, L. K. Chan, A. T. Schneider, L. Küsgens, F. Böhm, Y. Boege, M. E. Healy, J. Schmitt, S. Comtesse, M. Castoldi, C. Preisinger, M. Szydlowska, E. Focaccia, N. T. Gaisa, S. H. Loosen, S. Jörs, F. Tacke, C. Roderburg, V. Keitel, J. G. Bode, P. Boor, R. J. Davis, T. Longerich, F. Geisler, M. Heikenwalder, A. Weber, M. Vucur, T. Luedde, JNK signaling prevents biliary cyst formation through a CASPASE-8-dependent function of RIPK1 during aging. Proc. Natl. Acad. Sci. U.S.A. 118, e2007194118 (2021). 33798093 10.1073/pnas.2007194118 PMC8000530

[32] J. He, M. Gerstenlauer, L. K. Chan, F. Leithäuser, M. M. Yeh, T. Wirth, H. J. Maier, Block of NF-kB signaling accelerates MYC-driven hepatocellular carcinogenesis and modifies the tumor phenotype towards combined hepatocellular cholangiocarcinoma. Cancer Lett. 458, 113–122 (2019). 31128214 10.1016/j.canlet.2019.05.023

[33] M. J. Wolf, A. Adili, K. Piotrowitz, Z. Abdullah, Y. Boege, K. Stemmer, M. Ringelhan, N. Simonavicius, M. Egger, D. Wohlleber, A. Lorentzen, C. Einer, S. Schulz, T. Clavel, U. Protzer, C. Thiele, H. Zischka, H. Moch, M. Tschöp, A. V. Tumanov, D. Haller, K. Unger, M. Karin, M. Kopf, P. Knolle, A. Weber, M. Heikenwalder, Metabolic activation of intrahepatic CD8+ T cells and NKT cells causes nonalcoholic steatohepatitis and liver cancer via cross-talk with hepatocytes. Cancer Cell 26, 549–564 (2014). 25314080 10.1016/j.ccell.2014.09.003

[34] Z. Strizova, I. Benesova, R. Bartolini, R. Novysedlak, E. Cecrdlova, L. K. Foley, I. Striz, M1/M2 macrophages and their overlaps – Myth or reality? Clin. Sci. (Lond.) 137, 1067–1093 (2023). 37530555 10.1042/CS20220531 PMC10407193

[35] C. Wang, C. Ma, L. Gong, Y. Guo, K. Fu, Y. Zhang, H. Zhou, Y. Li, Macrophage polarization and its role in liver disease. Front. Immunol. 12, 803037 (2021). 34970275 10.3389/fimmu.2021.803037 PMC8712501

[36] T. M. de Almeida, R. C. Leitão, J. D. Andrade, W. Beçak, F. J. Carrilho, S. Sonohara, Detection of micronuclei formation and nuclear anomalies in regenerative nodules of human cirrhotic livers and relationship to hepatocellular carcinoma. Cancer Genet. Cytogenet. 150, 16–21 (2004). 15041218 10.1016/j.cancergencyto.2003.08.001

[37] H. Dungrawala, K. L. Rose, K. P. Bhat, K. N. Mohni, G. G. Glick, F. B. Couch, D. Cortez, The replication checkpoint prevents two types of fork collapse without regulating replisome stability. Mol. Cell 59, 998–1010 (2015). 26365379 10.1016/j.molcel.2015.07.030 PMC4575883

[38] Y. Zhao, L. Yu, S. Zhang, X. Su, X. Zhou, Extrachromosomal circular DNA: Current status and future prospects. eLife 11, e81412 (2022). 36256570 10.7554/eLife.81412 PMC9578701

[39] L. Mohr, E. Toufektchan, P. von Morgen, K. Chu, A. Kapoor, J. Maciejowski, ER-directed TREX1 limits cGAS activation at micronuclei. Mol. Cell 81, 724–738.e9 (2021). 33476576 10.1016/j.molcel.2020.12.037 PMC7897315

[40] S. T. Sin, J. Deng, L. Ji, M. Yukawa, R. W. Chan, S. Volpi, A. Vaglio, P. Fenaroli, P. Bocca, S. H. Cheng, D. K. Wong, K. O. Lui, P. Jiang, K. C. A. Chan, R. W. Chiu, Y. M. D. Lo, Effects of nucleases on cell-free extrachromosomal circular DNA. JCI Insight 7, e156070 (2022). 35451374 10.1172/jci.insight.156070 PMC9089787

[41] E. Pikarsky, R. M. Porat, I. Stein, R. Abramovitch, S. Amit, S. Kasem, E. Gutkovich-Pyest, S. Urieli-Shoval, E. Galun, Y. Ben-Neriah, NF-κB functions as a tumour promoter in inflammation-associated cancer. Nature 431, 461–466 (2004). 15329734 10.1038/nature02924

[42] Z. Dou, K. Ghosh, M. G. Vizioli, J. Zhu, P. Sen, K. J. Wangensteen, J. Simithy, Y. Lan, Y. Lin, Z. Zhou, B. C. Capell, C. Xu, M. Xu, J. E. Kieckhaefer, T. Jiang, M. Shoshkes-Carmel, K. M. A. A. Tanim, G. N. Barber, J. T. Seykora, S. E. Millar, K. H. Kaestner, B. A. Garcia, P. D. Adams, S. L. Berger, Cytoplasmic chromatin triggers inflammation in senescence and cancer. Nature 550, 402–406 (2017). 28976970 10.1038/nature24050 PMC5850938

[43] S. Glück, B. Guey, M. F. Gulen, K. Wolter, T.-W. Kang, N. A. Schmacke, A. Bridgeman, J. Rehwinkel, L. Zender, A. Ablasser, Innate immune sensing of cytosolic chromatin fragments through cGAS promotes senescence. Nat. Cell Biol. 19, 1061–1070 (2017). 28759028 10.1038/ncb3586 PMC5826565

[44] H. Yang, H. Wang, J. Ren, Q. Chen, Z. J. Chen, cGAS is essential for cellular senescence. Proc. Natl. Acad. Sci. U.S.A. 114, E4612–E4620 (2017). 28533362 10.1073/pnas.1705499114 PMC5468617

[45] H. Liu, H. Zhang, X. Wu, D. Ma, J. Wu, L. Wang, Y. Jiang, Y. Fei, C. Zhu, R. Tan, P. Jungblut, G. Pei, A. Dorhoi, Q. Yan, F. Zhang, R. Zheng, S. Liu, H. Liang, Z. Liu, H. Yang, J. Chen, P. Wang, T. Tang, W. Peng, Z. Hu, Z. Xu, X. Huang, J. Wang, H. Li, Y. Zhou, F. Liu, D. Yan, S. H. E. Kaufmann, C. Chen, Z. Mao, B. Ge, Nuclear cGAS suppresses DNA repair and promotes tumorigenesis. Nature 563, 131–136 (2018). 30356214 10.1038/s41586-018-0629-6

[46] C. Zierhut, N. Yamaguchi, M. Paredes, J. D. Luo, T. Carroll, H. Funabiki, The cytoplasmic DNA sensor cGAS promotes mitotic cell death. Cell 178, 302–315.e23 (2019). 31299200 10.1016/j.cell.2019.05.035 PMC6693521

[47] G. Dunphy, S. M. Flannery, J. F. Almine, D. J. Connolly, C. Paulus, K. L. Jønsson, M. R. Jakobsen, M. M. Nevels, A. G. Bowie, L. Unterholzner, Non-canonical activation of the DNA sensing adaptor STING by ATM and IFI16 mediates NF-κB signaling after nuclear DNA damage. Mol. Cell 71, 745–760.e5 (2018). 30193098 10.1016/j.molcel.2018.07.034 PMC6127031

[48] M. Charni-Natan, I. Goldstein, Protocol for primary mouse hepatocyte isolation. STAR Protoc. 1, 100086 (2020). 33111119 10.1016/j.xpro.2020.100086 PMC7580103

[49] G. Toda, T. Yamauchi, T. Kadowaki, K. Ueki, Preparation and culture of bone marrow-derived macrophages from mice for functional analysis. STAR Protoc. 2, 100246 (2021). 33458708 10.1016/j.xpro.2020.100246 PMC7797923

