# Supplementary material for: Extrachromosomal circular DNA promotes inflammation and hepatocellular carcinoma development
Source: Sci Adv. 2025 Oct 17;11(42):eadw0272. doi: 10.1126/sciadv.adw0272 (PMC12533639; doi:10.1126/sciadv.adw0272)
Supplement: Supplementary file 1 — Supplementary Text Figs. S1 to S11 Tables S1 to S7 References [file sciadv.adw0272_sm.pdf]

Science Advances
AAAS

Supplementary Materials for

Extrachromosomal circular DNA promotes inflammation and hepatocellular carcinoma development

Lap Kwan Chan *et al.*

Corresponding author: Achim Weber, achim.weber@usz.ch; Lap Kwan Chan, lapkwan.chan@usz.ch

Sci. Adv. **11**, eadw0272 (2025)
DOI: 10.1126/sciadv.adw0272

This PDF file includes:

Supplementary Text
Figs. S1 to S11
Tables S1 to S7
References

Supplementary Text

Extended Methods

Cell lines

AML12 (CRL-2254), HEK293T (CRL-3216), and HeLa (CCL-2) cells were obtained from ATCC. Mycoplasma, bacterial, and fungal testing was performed by the supplier with the information that none of these contaminants were detected.

Serum enzyme activities measurement

To determine the serum ALT and AST activities, approximately 50 μl of blood was collected from the tail vein of mice at the indicated time points using BD Microtainer SST Tubes (BD #365968). After settling at room temperature for at least 10 minutes, serum was obtained by centrifugation at 8500g for 90 seconds. ALT and AST activities were measured using Reflotron Plus (Roche) with GOT/AST and GPT/ALT stripes (Roche #10745120202 & 10745138202).

Histology, immunohistochemistry (IHC), immunofluorescence (IF) and microscopy

For histological analysis, mice were euthanized and livers were harvested and fixed in 4% neutral buffered formalin (PFA) for at least 24 hours. Liver tissues were then dehydrated and embedded with paraffin. Paraffin sections of 3μm thickness were used in IHC and IF staining.

Hematoxylin and eosin (H&E) stains were performed by a standard protocol. For IHC, immune stains were performed by Leica Bond RX. Stained IHC slides were scanned by Hamamatsu C9600 slide scanner. For IF, stains were performed manually, using heat-mediated antigen retrieval

(pressure cooker, 10 minutes) with citrate buffer (pH 6) after deparaffinization and rehydration. Tissue sections were blocked with 5% BSA/PBS solution for one hour at room temperature prior to overnight primary antibody incubation, followed by one hour of fluorophore-tagged secondary antibody incubation at room temperature. Slides were mounted with EverBrite hardset mounting medium carrying DAPI (Biotium #23004) for microscopy. IF images were acquired with Leica DM6 microscope or Leica SP8 confocal microscope. Images were analyzed using QuPath (version 0.4.4) or ImageJ (Fiji). A list of IHC and IF antibodies is provided in Table S4.

Western blot

Liver tissues were harvested and snap-frozen followed by homogenization through powderization. Total proteins were extracted with 4% sodium dodecyl sulfate (SDS)/100mM Tris-HCl. Protein concentration was determined by the BCA Protein Assay Kit (Thermo Scientific #23228). For Western blots, protein lysates (10-20μg for tissue lysates; 5μg for cell lysates) were prepared with NuPage LDS Sample Buffer (Invitrogen #NP0007) and 4-20% mini-PROTREAN TGX gels (Bio-rad #4568096 and #4568094) were used to separate proteins. Protein were transferred onto PVDF membranes using the Transblot-turbo system (Bio-Rad). Membranes were blocked with 5% BSA in TBS buffer/0.1% Tween-20 or 1X ROTI Block (ROTH #A151.2) for 1 hour. Primary and secondary antibodies were then applied. Signals were detected by Fushion Solo. Densitometry of bands was performed using ImageJ. A list of Western blot antibodies is provided in Table S5.

Primary hepatocyte and immune cell isolation from the liver

Hepatocyte isolation was performed according to the proposed protocol (48). Briefly, mice were first anesthetized by ketamine (100mg/kg) and xylazine (16mg/kg). A pre-warmed (37°C) pre-

perfusion solution (0.5mM EDTA/20mM HEPES/HBSS) was perfused via the vena cava using a constant flow rate of 7ml/min regulated by a peristaltic pump (VWR). Once yellow spots were seen in the liver, the portal vein was cut to release the blood and perfusion buffer. The liver was first perfused with the pre-perfusion buffer for 5 minutes and then switched to perfusion solution (20mM HEPES / 1X Penicillin/Streptomycin / 3mM $CaCl_2$ / DMEM/F12 / 0.2mg/ml Liberase (Roche #05401127001)) for 10 minutes. At the end of the liberase digestion, the liver was carefully transferred to a 10cm petri dish and washed with Wash solution (4% FBS / 1X Penicillin/Streptomycin / Willian's E medium) and the gall bladder was removed. The liver was then transferred to a new petri dish with 10ml of Wash solution and hepatocytes were gently released from the liver using forceps. The Wash solution containing cells was filtered through a 70μm cell strainer (Falcon #352350) and stored on ice. The cell suspension was centrifuged at 20g for 3 minutes. The pellet was used for hepatocyte isolation and the supernatant, which contained non-parenchymal cells, was transferred to a new 50ml Falcon tube for immune cell isolation. For hepatocyte purification, the pellet was resuspended with 10ml of Wash solution and mixed with 10ml of 90% percoll solution (Cytiva #GE-17-0891-01) and centrifuged at 200g for 10 minutes. This step was repeated one more time to increase the purity of viable cells. The pellet was then resuspended in Wash solution and seeded at a density of $3x10^5$ cells/well in a collagen pre-coated 24-well plate. The medium was exchanged to Hepatocyte medium (Wash solution / 50ng/ml EGF / 1μg/ml insulin / 10μg/ml Transferrin / 1.3μg/ml Hydrocortisone) after 4 hours. A lower seeding density was used for hepatocytes in the DNA fiber assay. For isolating immune cells, the volume was brought to 50ml using the Wash solution. The solution was centrifuged at 20g for 3 minutes and the supernatant was transferred to a new 50ml Falcon tube. This step was repeated for additional two times. The transferred supernatant was then centrifuged at 2000 rpm for 5 minutes.

The immune cell pellet was resuspended in 10ml of 36% Percoll solution and centrifuged at 2000rpm for 20 minutes at 4°C. RBCs were lysed with 1X RBC lysis buffer (G Biosciences #786-649) at room temperature for 5 minutes. The immune cells were washed once with 10ml of PBS and pelleted followed by snap-freezing and storage.

Preparation of bone-marrow-derived macrophages (BMDM)

BMDM was prepared according to the proposed protocol (49). Briefly, mice were euthanized using CO_2. The femur and tibia from both limbs were collected in 1.5ml Eppendorf tubes and stored on ice. Under a cell culture hood, 1ml of cold PBS was carefully injected through the collected femur and tibia to flush out the bone marrow cells into a well of a 6-well plate. The collected bone marrow cells were centrifuged at 200g for 5 minutes at 4°C. The cell pellet was resuspended in 1X RBC lysis buffer and incubated at room temperature for 5 minutes to remove erythrocytes. The cells were then washed once with 10ml of cold PBS and pelleted. The cell pellet was then resuspended in 12 ml of Bone Marrow Culture Medium (DMEM / 10% FBS/ 1% Penicillin/Streptomycin / 10ng/ml M-CSF) and distributed into a 6-well plate, with 2ml per well. Each well carried approximately 3 million cells. On day 4, half of the medium was exchanged with fresh Bone Marrow Culture Medium. BMDMs were ready for experiments on day 7.

Transfection of poly(dGdC), linear gDNA and eccDNA

For generating linear gDNA, total gDNA was isolated from AML12 cells and HeLa cells. An aliquot of the gDNA (1µg) was sonicated using Bandelin SONOPLUS Mini 20 for 50 seconds. The size of DNA fragments was confirmed to be below 3kb by gel electrophoresis. Transfections of poly(dGdC) (InvivoGen #tlrl-pgcn), linear gDNA, and eccDNA were performed by

Lipofectamine 3000 Transfection Reagent (Invitrogen #L3000008) according to the manufacturer's instruction. Transfected BMDMs were collected 12 hours after the transfection. Controls received only transfection reagents.

Flow cytometry

For detection of apoptotic cells, cells were treated with staurosporine (1μM) (MedChemExpress #HY-15141) for 24 hours or reversine (0.5μM) (MedChemExpress # HY-14711) for 48 hours. Cells were trypsinized and washed twice with cold PBS. Pelleted cells were resuspended in Annexin V binding buffer (BioLegend #640914). Approximately 1 million cells in a volume of 100μl were prepared in a 5ml test tube. Annexin V-FITC and Propidium Iodine were added to the cells followed by an incubation for 15 minutes at room temperature in the dark. At the end of incubation, 400μl of Annexin V binding buffer was added to the tube, and the cells were analyzed with BD FACS Canto II. A list of Western blot antibodies is provided in Table S6.

Quantitative real-time PCR (qPCR)

Total RNA was isolated from pulverized liver tissues or cells using the RNeasy mini kit or RNeasy micro kit (Qiagen #74004 and #74104). Complementary DNA (cDNA) synthesis was performed using a High Capacity cDNA Reverse Transcription Kit (Applied Biosystems #4368814) with the extracted mRNA from each sample. qPCR was performed using SYBR Green PCR master mix (Applied Biosystem #43-687-02) with ViiA7 Real-Time PCR system (Applied Biosystems). A list of primers is provided in Table S7.

Primary nuclei and micronuclei isolation

Purification of micronuclei and primary nuclei was performed following the protocol introduced by Toufektchan and Maciejowski (30). Briefly, AML12 (6 x 150mm plates) and HEK293T cells (3 x 150mm plates) were treated with reversine for 48 hours to promote micronuclei formation. On the day of the experiment, cells were harvested and incubated with cytochalasin B for 30 minutes at 37°C, then pelleted and resuspended in 5 ml of lysis buffer. The cell lysate was then mixed with an equal volume of 1.8M sucrose buffer. A sucrose gradient was prepared by first adding 15 ml of 1.6M sucrose buffer at the bottom of a 50 ml falcon tube followed by layering 20 ml of 1.8M sucrose buffer on top. The cell lysate/1.8M sucrose buffer mixture was laid on top of the prepared sucrose gradient and centrifuged at 950g for 20 minutes at 4°C. The top 2ml were discarded. The next 5 ml of the micronuclei-enriched fraction were transferred to another 50 ml falcon tube and washed with 20 ml of PBS supplemented with 1X protease inhibitor followed by centrifugation at 1500g for 20 minutes at 4°C. The supernatant was discarded, leaving only 1 ml for resuspending the pellet. The sample was filtered through the cell strainer of the FACS round-bottom tube (Falcon #352235). Hoechst 33342 stain (1μg/ml) (Life technology #H3570) and MitoView Green dye (Biotium #70054-T) (40nM) were added to the sample 10 minutes prior to FACS sorting. Primary nuclei and micronuclei were sorted according to the size and Hoechst 33342 intensity using BD FACSAria III. MitoView dye was used to minimize mitochondria contamination during sorting.

Stimulation of BMDM with DNA, conditioned media or MN-enriched fraction

BMDM from WT mice was prepared as described above. On Day 7, 30,000 of BMDMs were seeded in each well of a 96 well plate. On the day of experiment, 30ng/ml of AML12 linear DNA

or isolated AML12 eccDNA was added to each well. BMDMs were harvested after 12 hours of incubation and RNA was isolated for qPCR analysis.

For preparing conditioned media, AML12 cells were treated with reversine for 48 hours. The culture medium was exchanged with fresh medium at the end of 48 hours and the cells were cultured for another 22 hours. Conditioned medium was then collected and filtered with 0.2μm filter. Non-treated cells were used as control. Conditioned media were added to BMDM at a ratio of 1:2 with the BMDM medium. BMDMs were collected after 8 or 16 hours of incubation.

To prepare MN-enriched fraction, AML12 cells were treated with reversine followed by sucrose gradient centrifugation as described above in the MN isolation protocol. After the sucrose gradient centrifugation, the MN-enriched fraction was collected (5ml) and washed with 20ml of PBS supplemented with protease inhibitor. The sample was centrifuged at 1500g for 20 minutes. The supernatant was removed and the sample was washed with 48ml of cold PBS. The sample was centrifuged at 3000g for 10 minutes. The purity of MN in the sample was assessed by flow cytometry. For the stimulation of BMDMs, purified MN-enriched fraction was added at a ratio of 1:3 to the BMDM culture medium. BMDMs were harvested after 16 hours for analysis. PBS was applied to the cells as non-treated controls (NT).

<u>eccDNA isolation and CirSeq</u>

We used the eccDNA isolation protocol proposed by Wang et al., 2023 for purifying eccDNA for transfection experiments and electron microscopy (22). Briefly, AML12 cells or HeLa cells were treated with 1μM staurosporine for 24 hours or with 0.5μM reversine for 48 hours. Cells were

collected and washed twice with PBS. The cells were pelleted and resuspended in 10ml of suspension buffer (10mM EDTA pH8 / 150 mM NaCl / 1% glycerol / lysis blue / RNase A / β-mercaptoethanol). 10 ml of Pyr buffer (0.5M pyrrolidine / 20mM EDTA / 1% SDS / β-mercaptoethanol / pH 11.8) was added to the resuspended cells and gently mixed well. The mixture was incubated at room temperature for 5 minutes. 10 ml of Buffer S3 (Qiagen Plasmid Plus Midi kit #12943) was then added to the solution and mixed well until the solution turned white. The mixture was centrifuged at 3148g for 20 minutes at 4°C. The clear lysate was filtered through the QIAfilter Cartridge (Qiagen Plasmid Plus Midi kit) and mixed with 1/3 volume of Buffer BB. Using QIAvac 24 plus, all lysates were passed through the QIAGEN Plasmid Plus spin column. The spin columns were then washed with ETR buffer and PE buffer. Crude circular DNA extract was eluted with 100μl of DNase-free water. The crude circular DNA concentration was determined by Qubit 1X dsDNA HS Assay Kit (invitrogen #Q33230). Next, plasmid-safe DNase (Lucigen #E3101K) with a final concentration of 0.4U/μl (1X Plasmid-safe Reaction Buffer / 1mM ATP / 10U Plasmid-safe DNase) was mixed with 3μg of crude circular DNA to eliminate the contaminating linear DNA. PacI was added to facilitate the elimination of mitochondrial DNA. The digested DNA was then cleaned up using the phenol/chloroform/isoamyl alcohol method. DNA was then precipitated from the aqueous fraction by adding 1μl of glycogen, 1/10 volume of sodium acetate (3M, pH5.5), and 3 volumes of 200 proof ethanol with incubation at -80°C for at least 3 hours. The precipitated DNA was centrifuged at 20,000g for 30 minutes at 4°C. The DNA pellet was washed once with 1 ml of freshly prepared 80% ethanol and resuspended in 50 μl of 2 mM Tris-HCl (pH 7). eccDNA was then selectively purified using Solution A (Bingene #220501). DNA resuspended in 2mM of Tris-HCl was mixed with 700μl of Solution A and incubated at room temperature for 5 minutes. The solution was then mixed well with 10μl of Dynabeads MyOne

Silane beads (Invitrogen #37002D) and put on a magnetic holder (Invitrogen DynaMag-2 #12321D). After settling for 2 minutes, all solution was discarded without disturbing the beads. The beads were washed twice with 300µl of Solution A using the same procedure. While leaving the tubes on the magnetic holder, the beads were washed twice with 700µl of 3.5M NaCl and twice with 800µl of freshly prepared 80% ethanol. eccDNA was eluted with 20µl of 0.1X elution buffer (Qiagen Plasmid Plus Midi kit).

For the purification of eccDNA from tissue, the isolation of crude circular DNA was performed using the QIAprep Spin Miniprep Kit (Qiagen # 27104). 50 mg of frozen tissue obtained from animals were cut into small pieces and digested overnight with proteinase K at 0.6U/µl (Thermo #EO0491) in 600µl of P1 solution (QIAprep Spin Miniprep Kit # 27104) at 50°C with shaking at 650rpm. The lysate was centrifuged at 1,000 rpm for 2 minutes and the supernatant was transferred to a 2ml eppendorf tube (DNase free) and LyseBlue was added at 1:1000 to the collected supernatant. 600µl of Buffer P2 was then added to the lysate and incubated at room temperature for 5 minutes. Immediately after the incubation, 840µl of Buffer N3 was added to neutralize the solution. The mixed solution was centrifuged at 13,000 rpm for 10 minutes at room temperature. 800µl of the supernatant was transferred to a QIAprep 2.0 spin column. The column was centrifuged for 30 seconds and the flow through was discarded. The column was then washed with 0.5ml of Buffer PB followed by 0.75ml of Buffer PE. The column was then dried by centrifuging at a maximum speed for 1 minute and the DNA was eluted with 100µl of DNase-free water. eccDNA purification was then performed as described above.

For preparing DNA samples for cirSeq, we used a different protocol that was suggested to preserve large circular DNA and reduce eccDNA loss due to the potential linearization of eccDNA by restriction enzymes (26). Briefly, high-molecular-weight (HMW) DNA was isolated from tissue using the MagAttract HMW DNA kit (Qiagen #67563) according to the manual of the manufacturer. Around 5-10 mg of tissue was obtained and briefly washed with cold PBS. The tissue was briefly spun down and resuspended in 220µl of Buffer ATL. 20µl of proteinase K was added to the sample and mixed well by vortexing. The sample was digested overnight at 56°C with shaking at 900rpm. After digestion, 200µl of the samples were transferred to a new 2ml Eppendorf tube. For each sample, 4µl of RNase A was added and incubated for 2 minutes at room temperature. Then, 150µl of Buffer AL 280µl of Buffer MB and 40µl of MagAttact Suspension G were added to each sample and incubated for 3 minutes at room temperature. Tubes were transferred to a Magnetic rack and waited for 1 minute until the beads were completely separated. Beads were washed twice with 700µl of Buffer MW1 by resuspending the beads and incubating at room temperature for 2 minutes followed by removal of the buffer using the magnetic rack. Beads were then washed twice with 700µl of Buffer PE. While keeping on the magnetic holder, the beads were then rinsed twice with 700µl of DNase-free water. Tubes were removed from the magnetic holder and the HMW DNA was eluted with 150µl of DNase-free water with shaking at 1400rpm for 3 minutes at room temperature. Purified HMW DNA was obtained by separating the beads from the elute using the magnetic holder. The eluted HMW DNA was transferred to a new 1.5ml DNA low-bind tube. Concentration of HMW DNA was determined by Qubit dsDNA HS Assay Kit (Invitrogen #Q32850).

Digestion of linear DNA was performed using plasmid-safe DNase (Lucigen #E3101K) with a final concentration of 0.2U/µl (1X Plasmid-safe Reaction Buffer / 1mM ATP / 20U Plasmid-safe DNase) for every 5µg of HMW DNA for 5 days at 37°C. For PN and MN samples, 50ng of DNA was used in this step. Plasmid-safe DNase and ATP were replenished every 24 hours. At the end of the 5-day digestion cycle, the DNase was inactivated at 70°C for 30 minutes. Rolling circle amplification was performed using the REPLI-g Mini Kit (Qiagen #150025) to amplify the circular DNA in the plasmid-safe DNase digested sample. In a 0.2ml PCR tube, 5µl of the digested HMW DNA was mixed with 5µl of Buffer D1 and incubated at 25°C for 3 minutes. After that, 10µl of Buffer N1 was added to the sample and mixed well by vortexing. 30µl of REPLI-g DNA polymerase master mix (29µl of REPLI-g Mini Reaction Buffer and 1µl of REPLI-g Mini DNA polymerase) were then added to each sample and incubated at 30°C for 16 hours. Sample were then inactivated at 65°C for 3 minutes. The quantity of double-stranded DNA was measured by Qubit BR dsDNA kit (Invitrogen #Q32850).

Library Preparation was performed on a 96-well plate using the NEBNext® Ultra™ II FS DNA Library Prep Kit for Illumina (NEB # E7805L). 20 ng of the RCA product from each sample was fragmented with 1.75µl of Ultra II FS Reaction Buffer and 0.5µl of Ultra II FS Enzyme Mix in a thermal cycler at 37°C for 25 minutes and 65°C for 30 minutes. Adaptor ligation was performed with 1µl of Adaptor (0.04 µl of adaptor diluted by 0.96 µl of 10 mM Tris-HCl, pH 7.5-8.0 with 10 mM NaCl), 7.5 µl of Ligation Master Mix and 0.25 µl of Ligation Enhancer. After 15 minutes of incubation at 20°C, 1 µl of diluted USER (0.75µl of USER with 0.25µl of nuclease-free water) was added and the samples were incubated at 37°C for 15 minutes. Adaptor-ligated DNA was cleaned with 0.8X (14.8 µl) of AMPure XP Beads from Beckman Coulter (A63881), mixed and

incubated for 5 minutes. Samples were kept on a magnetic holder (Invitrogen DynaMag™-96 Magnet # 12027). The supernatant was discarded, and beads were washed twice with 100 µl of 80 % ethanol while the plate was on the magnet. All ethanol was discarded, and the beads were dried for 2 minutes. DNA was eluted with 6µl of Elution Buffer (Qiagen Buffer EB #19086). 5µL of the eluted DNA from each well was transferred into a new 96-well plate.

PCR enrichment of 5µL adaptor-ligated DNA was performed by adding 2.5 µl of pre-mixed unique dual index primer pairs (NEBNext 96 unique dual index primer pairs kit 3 NEB #E6444L). Each well must contain a different primer pair for multiplexing. 6.25 µL of Ultra II Q5 Master Mix was added to each well. In a thermocycler, run a PCR program: 98°C for 30 seconds, 98°C for 10 seconds and 65°C for 75 seconds for 7 cycles and finish with 65°C for 5 minutes. The PCR reaction was cleaned using 10 µl (0.8X) of AMPure XP Beads from Beckman Coulter (A63881), mixed and incubated for 5 minutes. The supernatant was discarded without touching the beads and the beads were washed twice with 100 µl of 80 % Ethanol while the plate was on the magnetic holder (Invitrogen DynaMag™-96 Magnet # 12027). The beads were dried for 2 minutes, and DNA was eluted in 30 µl of Elution Buffer (Qiagen Buffer EB #19086) after 5 minutes of incubation. 28 µl of supernatant from each well was transferred to a new 96-well plate. The concentration of all samples was determined using Qubit 1X dsDNA HS Assay-Kit (ThermoFisher scientific # Q33231). Quality control of libraries was performed using TapeStation (Agilent High Sensitivity D1000 Reagents # 5067-5585 and High Sensitivity D1000 ScreenTape # 5067-5584). All samples were brought to the same concentration and 3 µl of each row of the 96-well plate was pooled into a 0.2 ml 8-tube strip. 10µl of each 0.2 ml tube was pooled into a 1.5 ml DNA LoBind tube. A cleanup step was performed with 64 µl (0.8X) of AMPure XP Beads in 80 µl of the pooled libraries

using a magnetic holder (DynaMag™-2 Magnet # 12321D). The beads were washed twice with 200 µl of 80 % ethanol using the magnetic holder. After drying for 2 minutes, samples were eluted in 20 µl of Elution Buffer (Qiagen Buffer EB #19086). 18µl of the eluted supernatant was transferred to a new DNA LoBind tube. The concentration of all samples was determined using Qubit 1X dsDNA HS Assay-Kit (ThermoFisher scientific # Q33231). A quality control step of libraries was performed again using TapeStation (Agilent High Sensitivity D1000 Reagents # 5067-5585 and High Sensitivity D1000 ScreenTape # 5067-5584). DNA libraries were submitted for 150bp pair-end Illumina sequencing using NovaSeq X Plus 10B.

To detect the presence of eccDNA, adapter sequences were trimmed from the reads using Trim Galore v. 0.6.10. The reads were then aligned either to the human reference genome hg38 or the mouse reference genome mm39 using the Burrows–Wheeler Aligner MEM v. 0.7.17 with default parameters. PCR and optical duplicates were removed with biobambam2 v. 2.0.183. The resulting BAM files were then analyzed to identify split reads and outward-facing discordant read pairs, which indicate circle-supporting reads. The coordinates of eccDNA were extracted from genomic regions enriched in these circle-supporting reads. The aligned reads were visualized using IGV version 2.17.2.

Design and replication of *in vivo* and *in vitro* experiments

Sample size required for this study was calculated based on the previous records of the status of liver damage in *Mcl1*$^{\Delta hep}$ mice (i.e. ALT and AST values). Blinding was not applicable since we aim to use all the possible animals with the desired genotypes. A stratified randomization strategy was used to maintain the same distribution of sex in the control and experimental groups.

Biological or technical replications were used when applicable. All n numbers in Fig. 1E, 1F, 2B, 2E, 3C, 3D, 5B, 5E, 5G, 5I, 5K, 6B, 6E, 6H, S2B, S2I, S3C, S10A, S10B and S11E represent biological replicates. All n numbers in Fig. 2G, 2H, 4A, 4L, S3I, S3J, S4C, S5B, S5C, S8E and S8F represent technical replicates.

Total RNAseq

Total RNA was extracted from frozen mouse tissue or frozen patient tissue using the RNeasy Micro kit (Quagen #74004). Total RNAseq using the Illumina Novaseq 6000 platform was performed at the Functional Genomic Center Zurich (FGCZ). Differential expression of genes and pathway analysis were performed by the FGCZ SUSHI platform. To perform GSEA analysis on the transcriptomic data, the datasets from WT, *Mcl1*$^{\Delta hep}$, and *Mcl1*$^{\Delta hep}$ *Sting1*$^{-/-}$ mice were imported to GSEA (4.3.3) Desktop Application and analyzed. For the preparation of heatmaps and statistical analysis of gene clusters, per-gene counts were transformed into transcripts per million (TPM) in $\log_2$ and then centered around zero using the per-gene row means. Small sets of key marker genes for cell types of interest (e.g., M1 and M2 macrophages) were generated using known lineage markers from the literature. For each cell type gene set, the mean-centered $\log_2$ TPM gene expression was calculated per sample. Global one-way ANOVA was performed, followed by post-hoc pairwise two-sided Student's t-tests.

Graphical illustrations

Graphical illustrations shown in the main figures (Figure 2A, 3A, 4E, 4H left panel and S8G) were created with BioRender.com.

Supplementary figures

Fig. S1

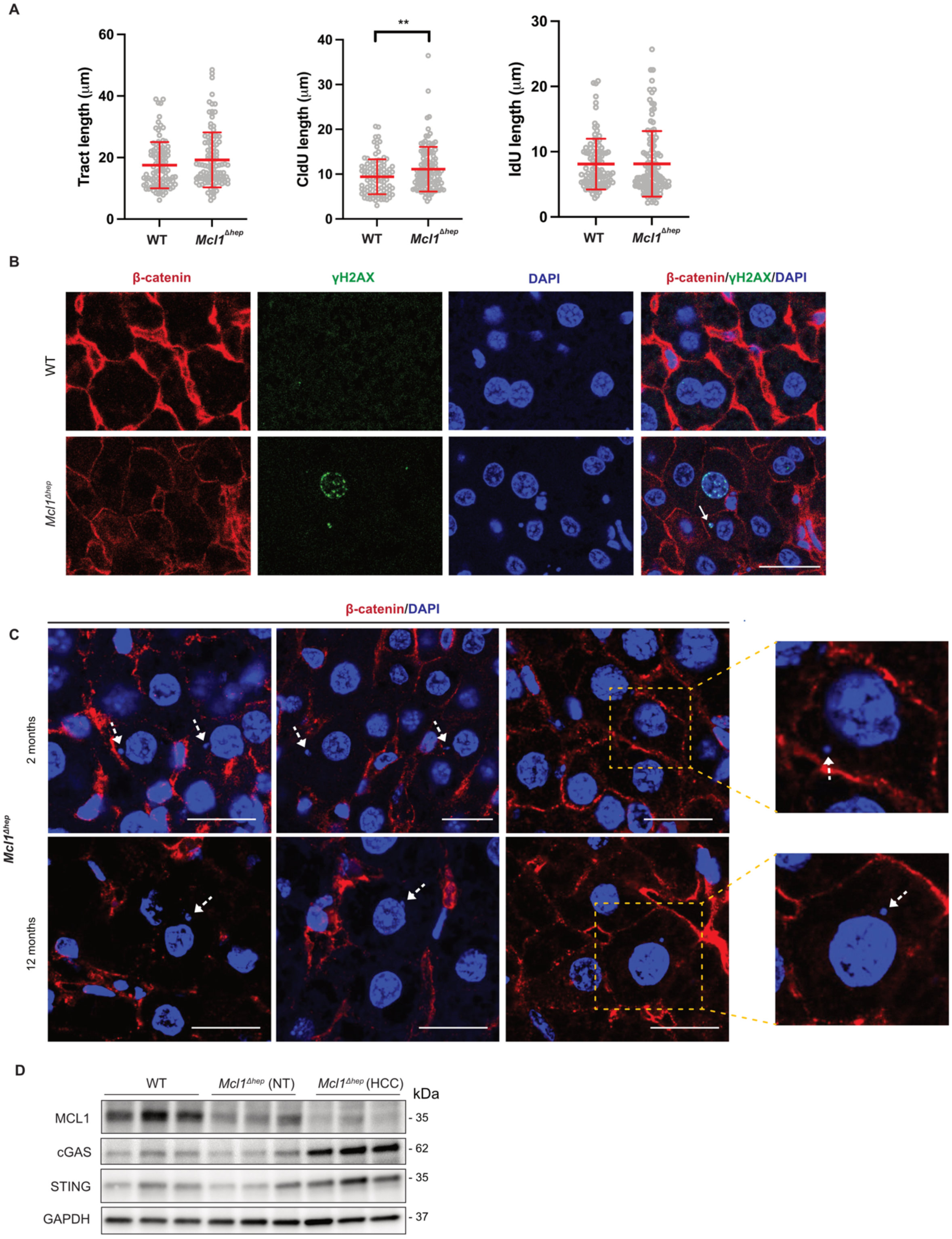

Fig. S1. A) DNA fiber assay on primary hepatocytes from WT and *Mcl1*$^{\Delta hep}$ mice after 24 hours in culture. CldU: 5-chloro-2'deoxyuridine; IdU: 5-Iodo-2'-Deoxyuridine. Quantification of CldU track length, IdU track length, and CldU+IdU total track length. Student's t-test: ** $p<0.01$. B) Immunofluorescence staining indicating micronuclei (arrow) showing DNA damage in 12-month-old liver tissue. Scale bar: 25µm. C) Further representative micronuclei (dashed arrow) observed in the hepatocytes of 2- and 12-month-old mice. Scale bar: 20 µm. D) Immunoblot of tissue extracts from the 12-month-old liver. WT: wild-type; NT: non-tumor; HCC: hepatocellular carcinoma.

Fig. S2

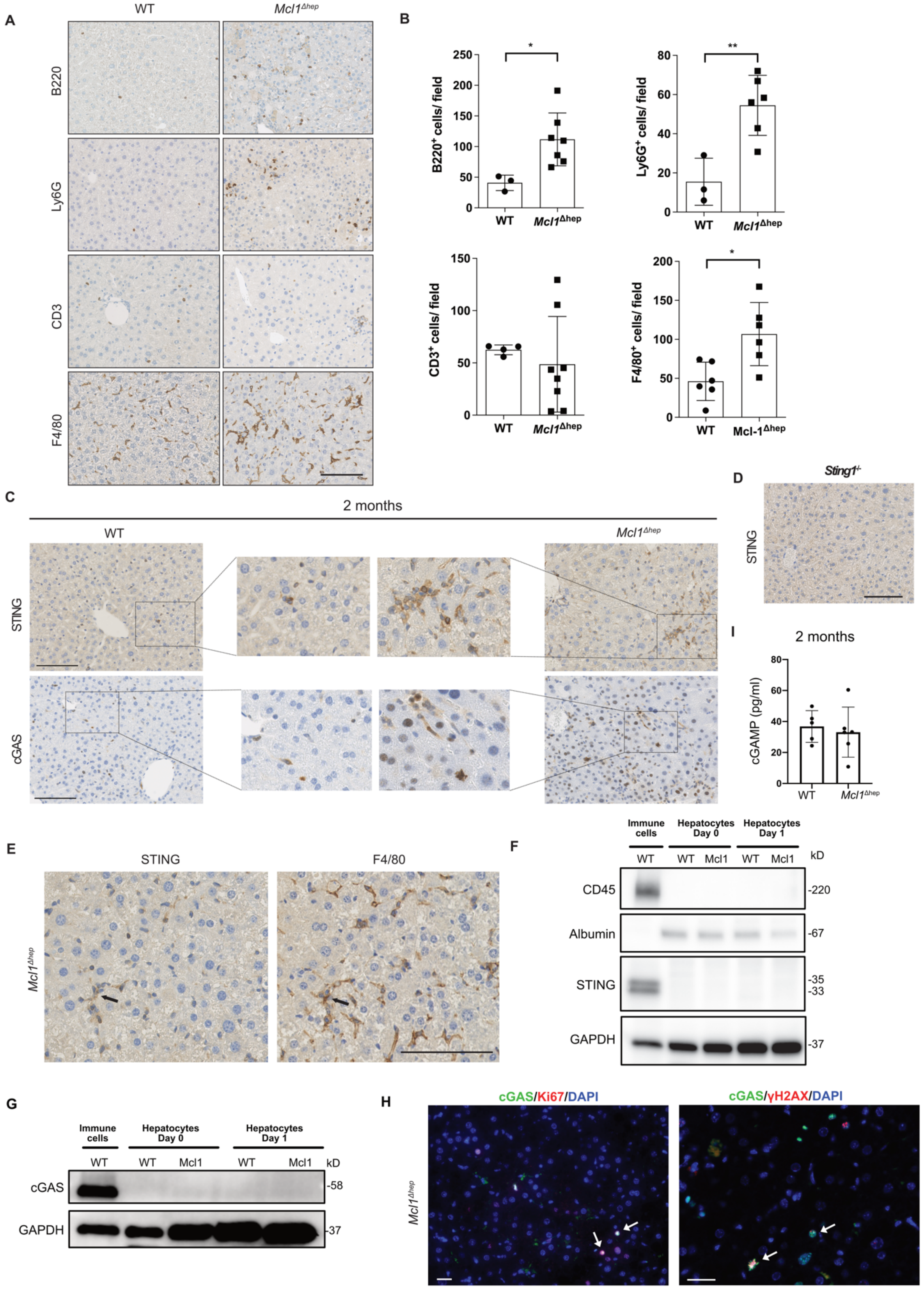

A
WT
Mcl1Δhep
B220
Ly6G
CD3
F4/80
B
B220+ cells/ field
Ly6G+ cells/ field
CD3+ cells/ field
F4/80+ cells/ field
WT
Mcl1Δhep
Mcl-1Δhep
C
2 months
STING
cGAS
D
Sting1-/-
STING
I
2 months
cGAMP (pg/ml)
E
STING
F4/80
F
Immune cells
Hepatocytes Day 0
Hepatocytes Day 1
WT
Mcl1
kD
CD45
Albumin
STING
GAPDH
G
cGAS
GAPDH
H
cGAS/Ki67/DAPI
cGAS/γH2AX/DAPI

Fig. S2. A) IHC staining of immune cell markers: B220, Ly6G, CD3, and F4/80. Scale bar: 50µm. B) Quantification of immune cells showing an increase in the infiltration of neutrophils, macrophages, and B cells. $n \geq 3$. Student's t-test: * $p<0.05$, ** $p<0.01$. C) IHC staining of STING and cGAS showing a different spatial expression pattern. Scale bar: 100µm. D) IHC staining of STING in *Sting1*$^{-/-}$ liver. Scale bar: 100µm. E) IHC staining of STING and F4/80 on serial sections showing the expression of STING and F4/80 in the same cells (arrow). Scale bar: 100µm. F&G) Western blot from isolated immune cells and hepatocytes from mouse livers. WT: wild-type; Mcl1: *Mcl1*$^{\Delta hep}$. Immune cells and hepatocytes (Day 0) were used directly after isolation. Hepatocytes were then kept in culture for 24 hours (Day 1), and protein was isolated. H) Immunofluorescence of cGAS/γH2AX or cGAS/Ki67 shows their colocalization in a subset of cells (arrows). Scale bar: 25µm. I) Measurement of cGAMP level from protein lysates using ELISA. $n \geq 5$.

Fig. S3

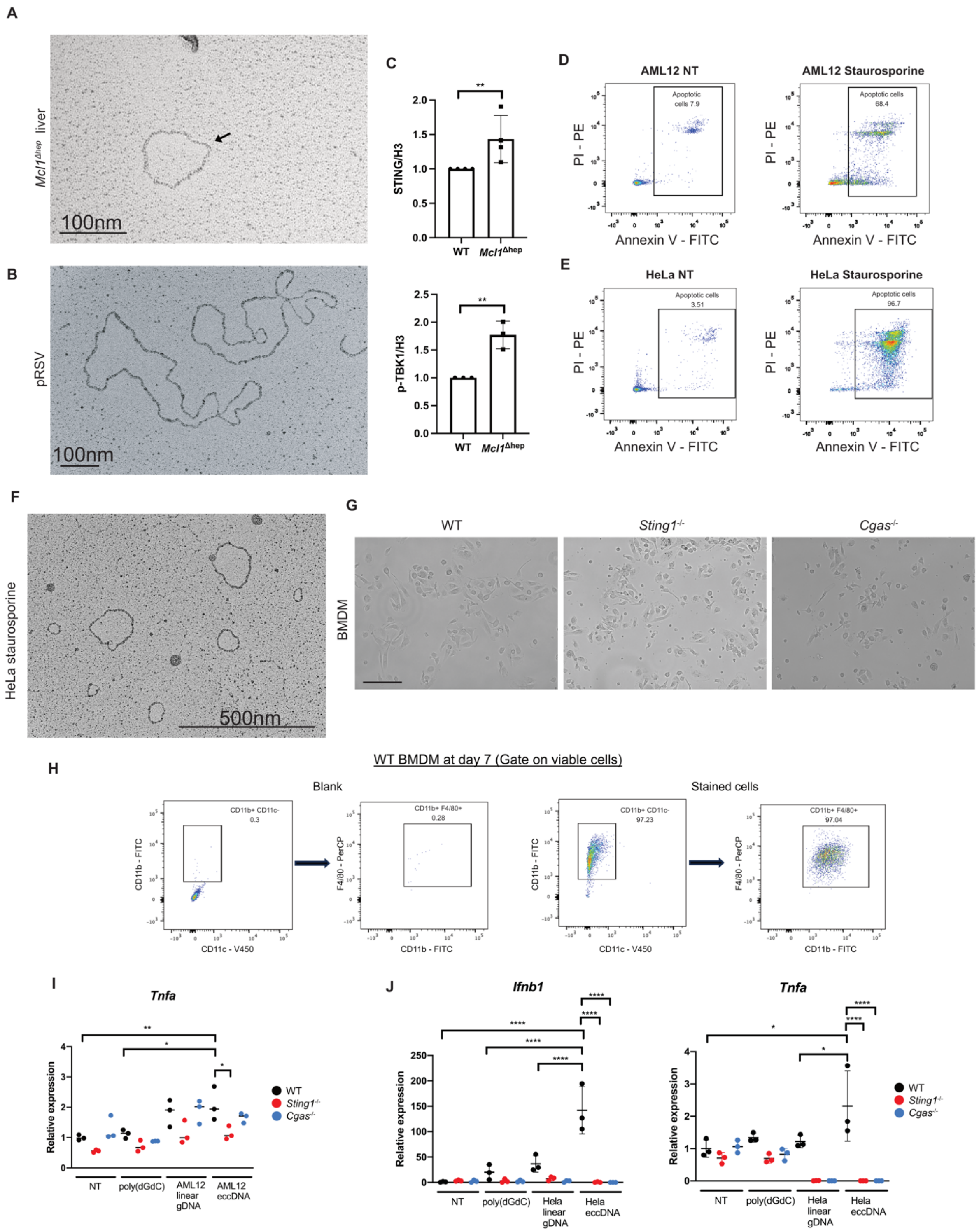

Fig. S3. A-B) Electron micrographs of eccDNA (arrow) from *Mcl1*$^{\Delta hep}$ liver tissue (A) and plasmid pRSV (B). Scale bar: 100nm. C) Densitometric analysis of Western blot for p-TBK1 and STING levels normalized to histone 3 (H3) level as a fold change compared to WT. n≥3. Student's

t-test ** p<0.01. D&E) Flow cytometry analysis of AML12 and HeLa cells treated with staurosporine for 24 hours. Cells were stained with Annexin V and PI for labelling early and late apoptotic stages. F) Visualization of eccDNA isolated from HeLa cells treated with 0.5M of staurosporine for 24 hours. Scale bar: 500nm. G) BMDM differentiated from bone marrow cells stimulated with M-CSF for 7 days. Scale bar: 50µm. H) Phenotyping of WT BMDM by flow cytometry. I) BMDMs from WT, *Sting1*$^{-/-}$, and *Cgas*$^{-/-}$ mice were transfected with 10ng/ml of AML12 eccDNA, AML12 linear genomic DNA, and poly(dGdC). *Tnfa* expression was analyzed 12 hours after transfection. n=3. J) BMDMs from WT, *Sting1*$^{-/-}$, and *Cgas*$^{-/-}$ mice were transfected with 100ng/ml of poly(dGdC), HeLa linear gDNA, or HeLa eccDNA. The expression of *Ifnb1* and *Tnfa* was detected by qPCR. n=3. One-way ANOVA: * p<0.05, ** p<0.01, *** p<0.001, **** p<0.0001.

Fig. S4

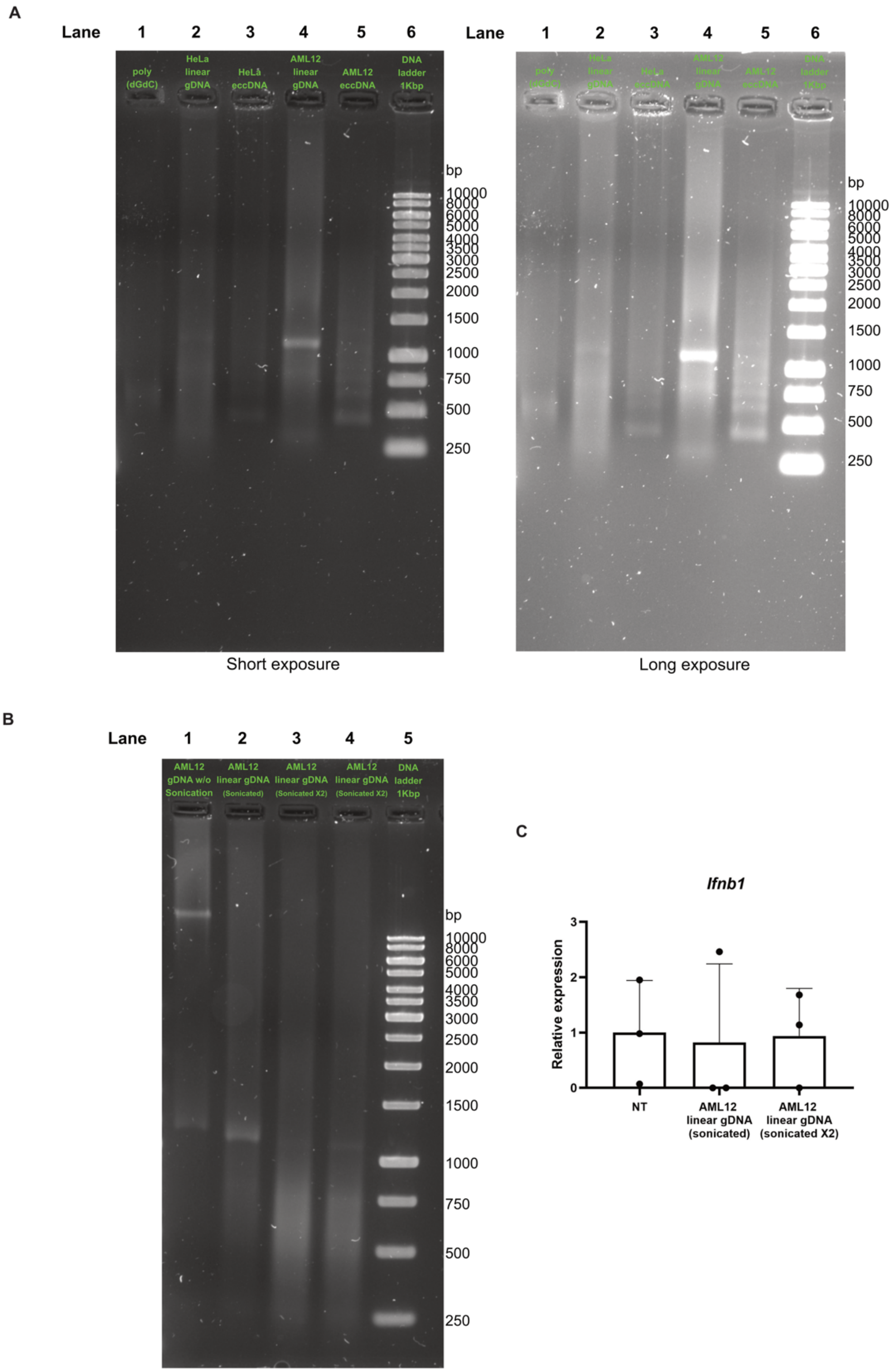

Fig. S4. A) Visualization of the molecular size of DNA used for stimulating bone marrow-derived macrophages (BMDMs). Agarose gel electrophoresis showing the molecular size of DNA. For each lane, 10ng of DNA was loaded onto a 1% agarose gel. Lane 1: poly(dGdC); lane 2: HeLa

linear DNA; lane 3: HeLa eccDNA; lane 4: AML12 linear gDNA; lane 5: AML12 eccDNA; lane 6: 1 Kbp DNA ladder. Left and right gel images were taken from the same gel with different exposure times. B) Study of the effect of additional shearing of linear gDNA in inducing *Ifnb1* in bone-marrow derived macrophages (BMDMs). Agarose gel electrophoresis is used to visualize the molecular size of DNA. Lane 1: AML12 linear gDNA without sonication; lane 2: sonicated AML12 linear gDNA (representing linear gDNA sample used in Fig. 2H); lane 3 & 4: repeated sonication of the linear DNA sample used in lane 2. C) Quantitative PCR analysis of *Ifnb1* expression in WT BMDMs. BMDM were transfected with 10ng of AML12 linear gDNA (lane 2) or AML12 linear gDNA with repeated sonication (lane 3). Note: The AML12 linear gDNA (lane 2) is the same sample used for the transfection experiment in Fig. 2H&S3I. NT: non-treated control. n=3.

Fig. S5

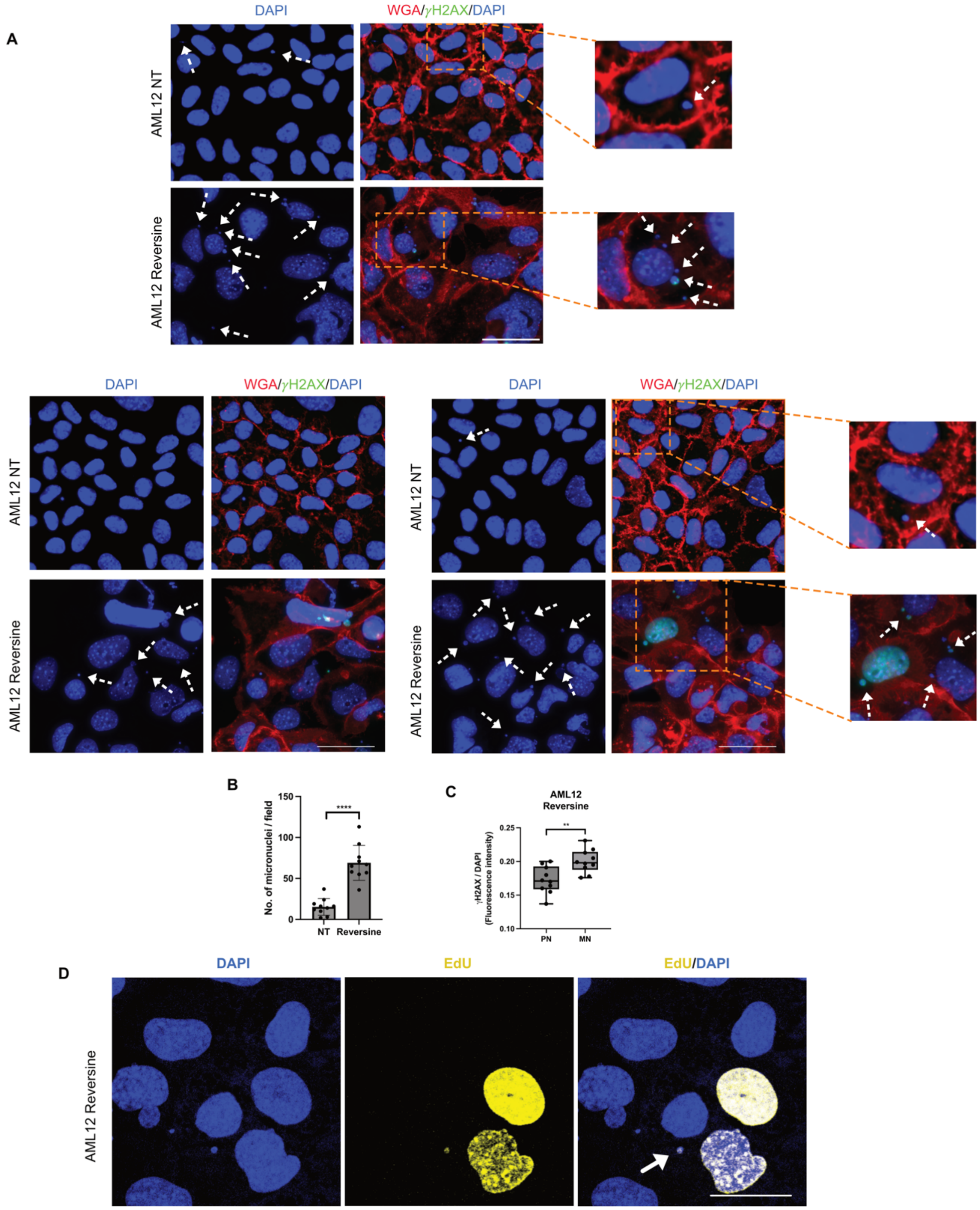

Fig. S5. A) AML12 cells treated with reversine for 48 hours showing an increase in micronuclei levels. NT: non-treated. WGA: wheat germ agglutinin (membrane dye). Three fields of view from the NT and reversine-treated cells are shown. Dashed arrow indicated micronuclei. Scale bar: 40μm. B) Quantification of the number of micronuclei observed per captured field. Student's t-test: **** $p<0.0001$. C) Comparison of γH2AX intensity normalized to DAPI intensity between

primary nuclei (PN) and micronuclei (MN). Student's t-test: ** $p<0.01$. D) AML12 cells were labeled with EdU for 2 hours at the end of the reversine treatment. Arrow indicates a S phase micronucleus. Scale bar: 20µm.

Fig. S6

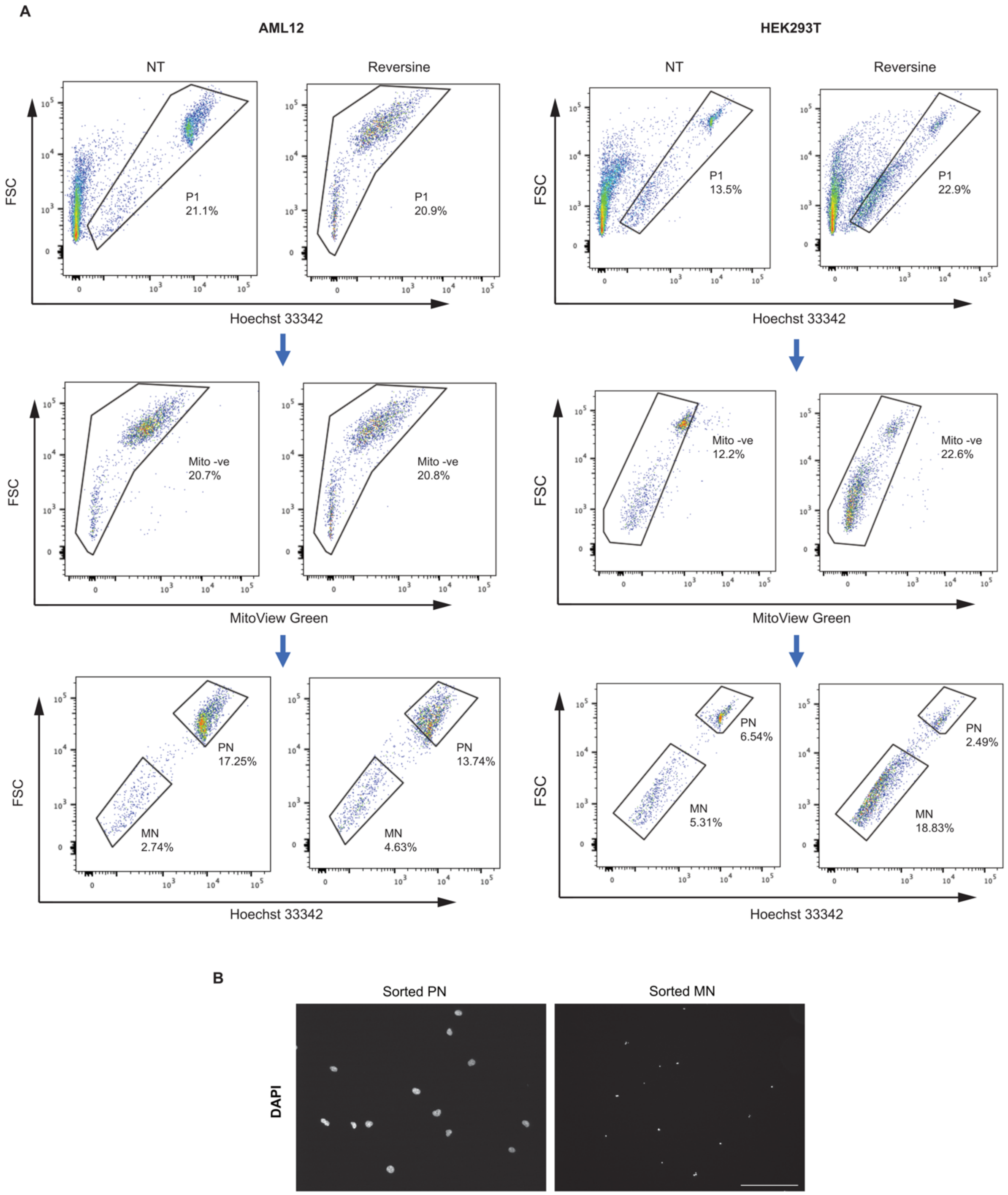

Fig. S6. A) FACS sorting of primary nuclei and micronuclei from AML12 cells and HEK293T cells 48 hours after reversine treatment. B) Images showing sorted primary nuclei (PN) and micronuclei (MN) from HEK293T cells. Scale bar: 100µm.

Fig. S7

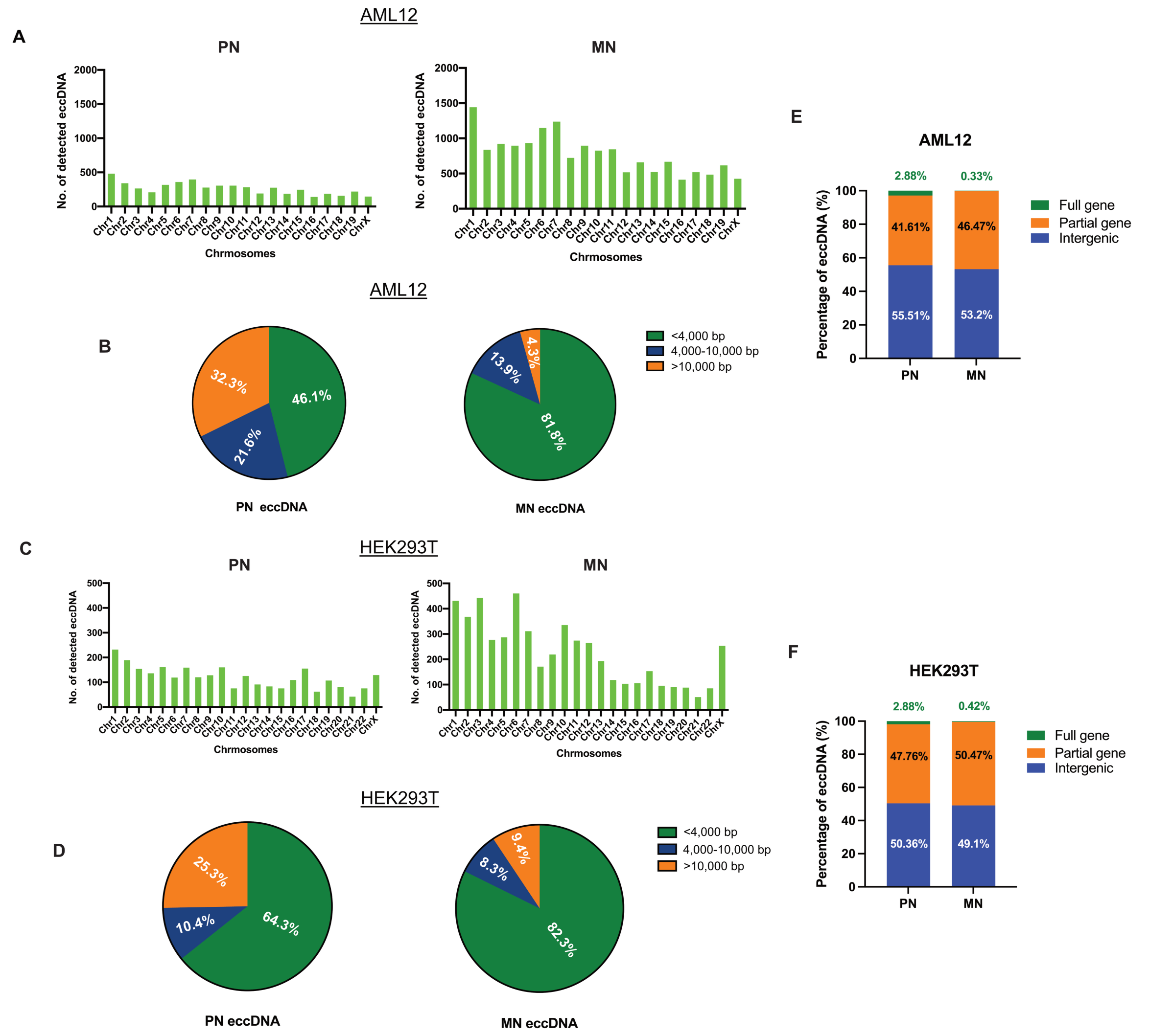

Fig. S7. A&C) eccDNA detected by cirSeq from isolated primary nuclei (PN) and micronuclei (MN) were mapped to the reference genome of mouse (AML12) and human (HEK293T). The frequency of detected eccDNA was plotted across all chromosomes. B&D) Pie charts showing the percentage of eccDNA with size <4,000bp, 4,000-10,000bp, and >10,000bp. E&F) Bar charts showing the percentages of eccDNA containing full gene, partial gene, and intergenic sequences in PN and MN.

Fig. S8

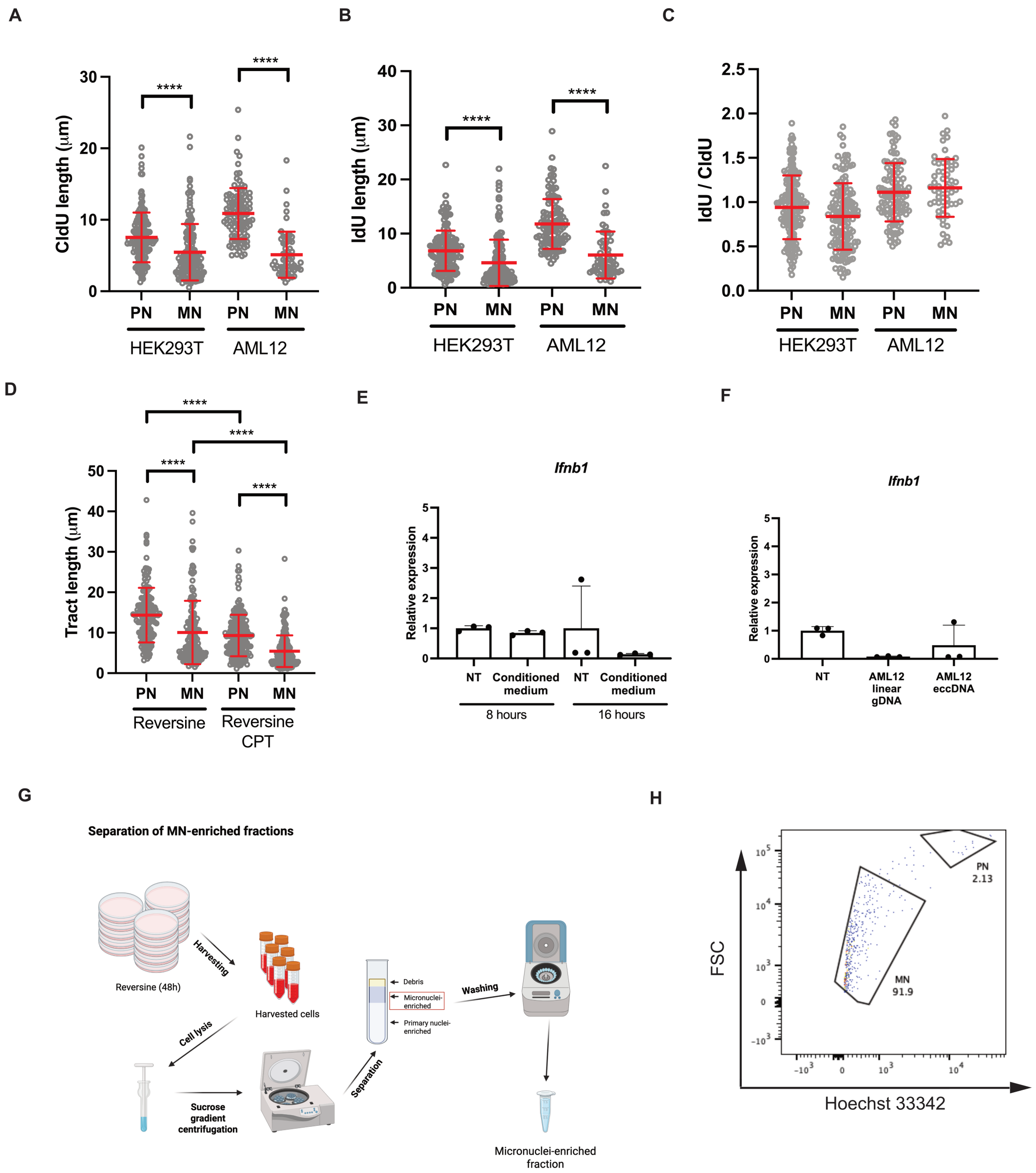

Fig. S8. A-C) NuSeF assay comparing the DNA fibers from primary nuclei (PN) and micronuclei (MN) of HEK293T and AML12 cells. CldU and IdU were pulse-labelled for 30 minutes each. The CldU tract length, IdU tract length, or IdU/CldU ratio was compared between PN and MN. Student's t-test: **** $p<0.0001$. D) Tract length (CldU + IdU) measurement from DNA fibers obtained from PN and MN from HEK293T cells treated with reversine with/without CPT. One-way ANOVA: **** $p<0.0001$. E) Quantitative PCR analysis of *Ifnb1* expression in BMDMs 8 or 16 hours after the treatment with conditioned medium from reversine-treated AML12 cells. n=3. F) Quantitative PCR analysis of *Ifnb1* expression in BMDMs 12 hours after the treatment of linear gDNA or eccDNA by adding DNA directly into the culture medium. NT samples were treated with PBS. n=3. G) Scheme of separation of the MN-enriched fraction. Created in BioRender.

Chan, L. (2025) https://BioRender.com/0omqdyj. H) Flow cytometry analysis of the MN-enriched fraction after additional washing steps.

Fig. S9

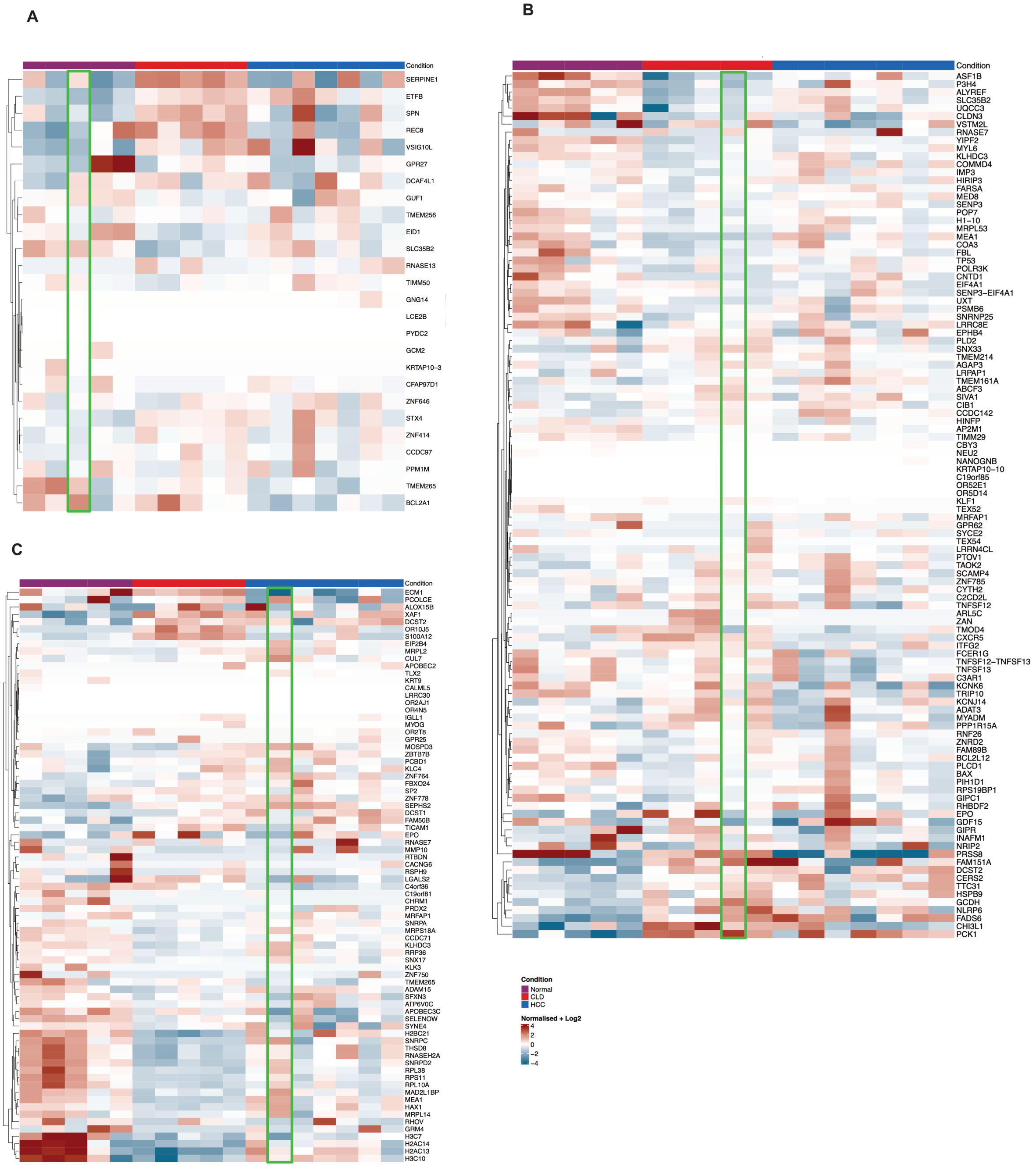

Fig. S9. Heatmap showing the expression of genes across normal liver, CLD, and HCC tissue. Gene lists show genes found in the eccDNA of A) normal liver, B) CLD, and C) HCC tissue. The samples with the list of genes present in eccDNA are highlighted by green rectangles.

Fig. S10

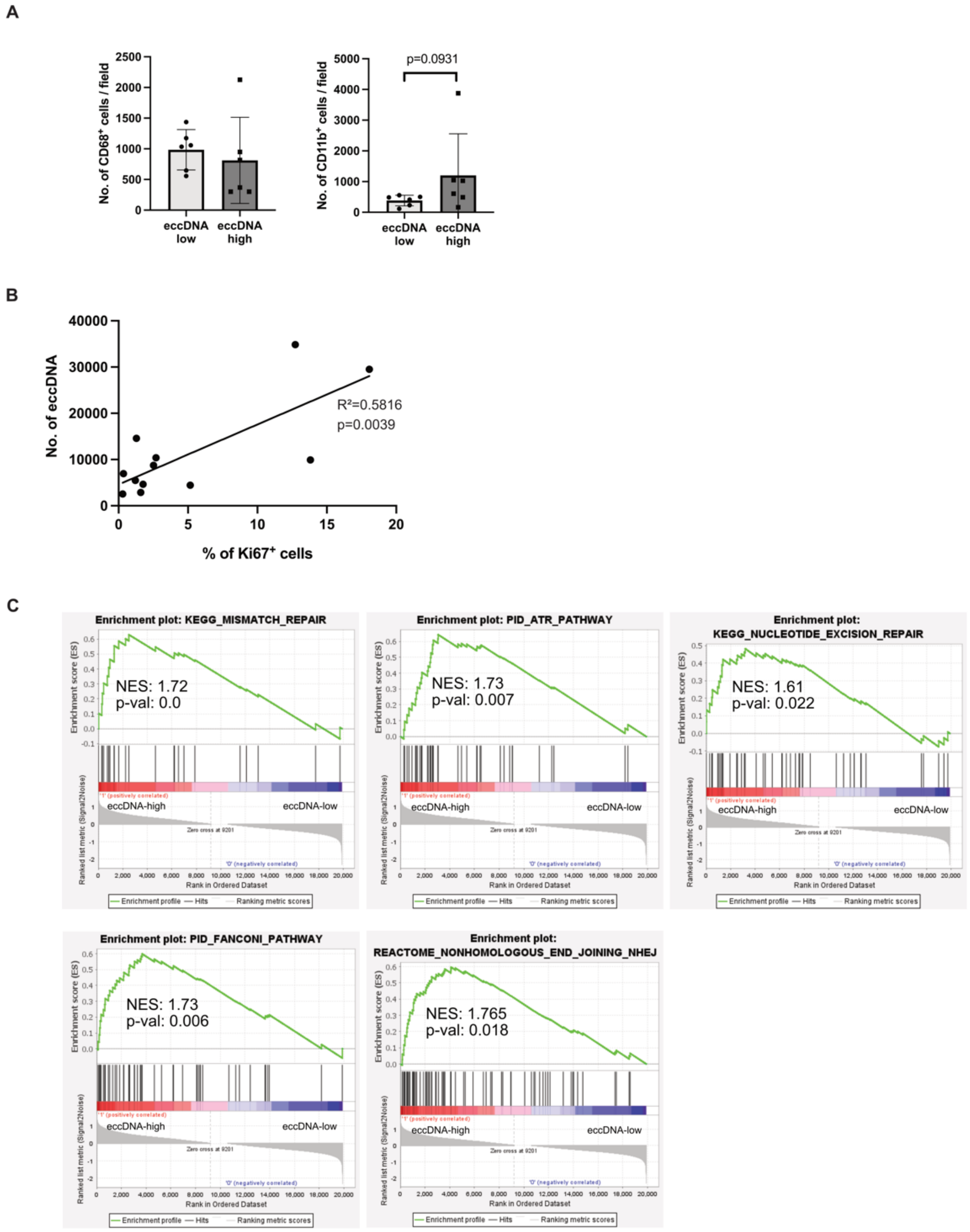

Fig. S10. A) Comparison of $CD68^{+}$ and $CD11b^{+}$ cells between eccDNA-high and eccDNA-low groups. Mann-Whitney test. n=6. B) Correlation of the percentage of $Ki67^{+}$ cells with the number of detected eccDNA in CLD and HCC tissue samples. n=12. C) GSEA analysis showing enrichment gene signatures related to DDR and DNA repair pathways.

Fig. S11

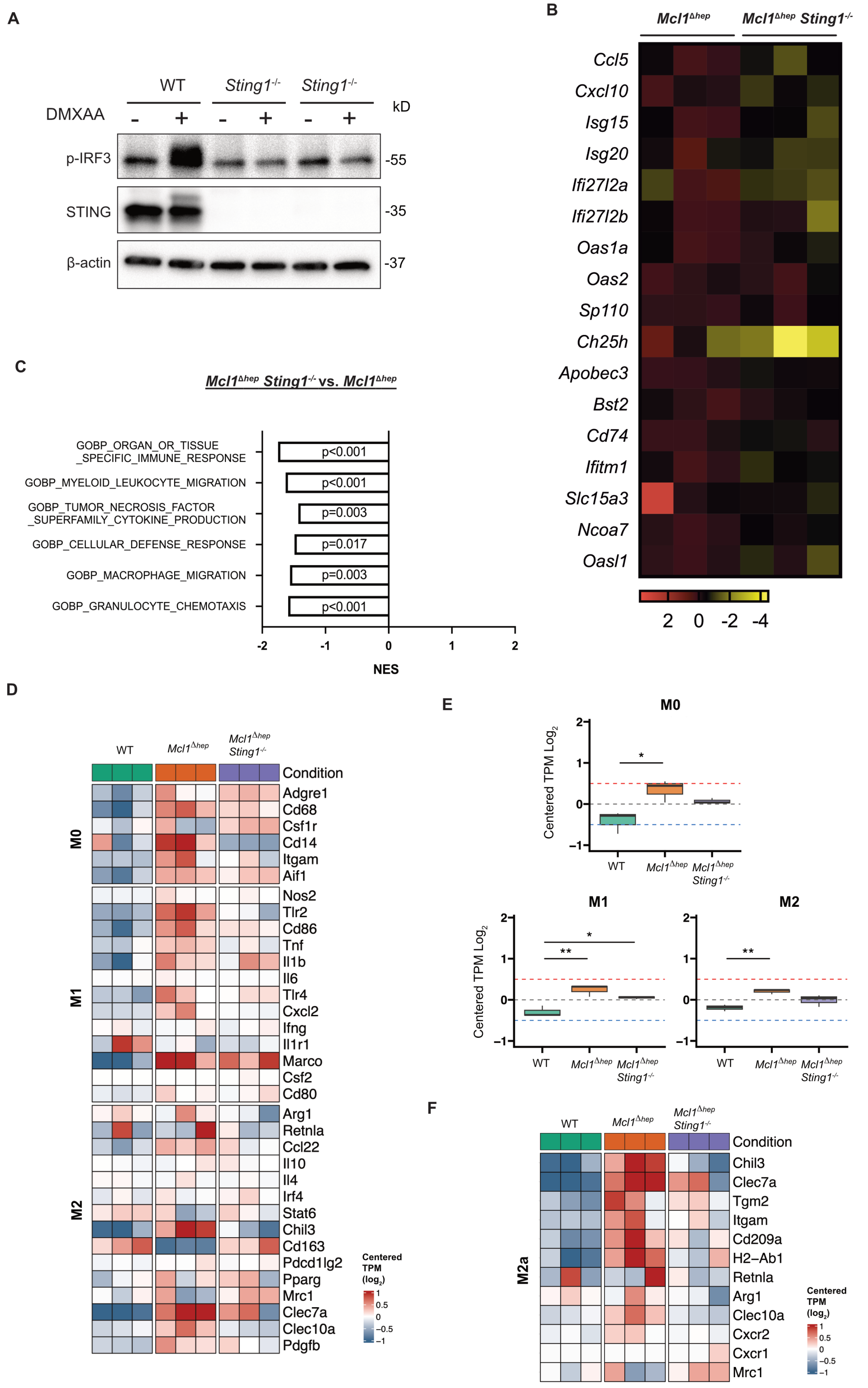

Fig. S11. A) Western blot for analyzing splenocytes obtained from WT and *Sting1*$^{-/-}$ mice. Splenocytes were treated with or without DMXAA for 3 hours. B) Heatmap of ISGs from RNAseq

data. C) Depleted GSEA signatures in *Mcl1*$^{\Delta hep}$ *Sting1*$^{-/-}$ mice. D) Heatmap showing M0-, M1-, and M2-associated genes extracted from RNAseq data of WT, *Mcl1*$^{\Delta hep}$, and *Mcl1*$^{\Delta hep}$ *Sting1*$^{-/-}$ mice. E) Boxplot comparing the log_2 centered TPM of the averaged M0-, M1-, and M2-associated genes. One-way ANOVA followed by post-hoc pairwise two-sided Student´s t-test: * $p<0.05$; ** $p<0.01$. F) Heatmap showing genes associated with the M2a subset of macrophages.

Supplementary Tables

	Total hepatocytes examined	Number of micronuclei^{+} hepatocytes	% of micronucleated hepatocytes
Mcl1$^{\Delta hep}$ #1	1000	53	5.3
Mcl1$^{\Delta hep}$ #2	1001	39	3.9
Mcl1$^{\Delta hep}$ #3	1003	18	1.8
Mcl1$^{\Delta hep}$ #4	1021	62	6.1
WT#1	1059	1	0.1
WT#2	1071	0	0
WT#3	1002	3	0.3
WT#4	1047	4	0.4

Table S1. Quantification of the percentage of micronucleated hepatocytes in 2-month-old WT and *Mcl1*$^{\Delta hep}$ mice.

sample_id	chr_circle	start_circle	end_circle	chr_gene	start_gene	end_gene	gene_symbol
WT1	chr1	88139927	88198043	chr1	88154708	88190011	*Mroh2a*
WT1	chr10	38696225	38697993	chr10	38696537	38697775	*Rfpl4b*
WT1	chr17	13093453	13097236	chr17	13094921	13096864	*Mrgprh*
WT1	chr17	13433078	13458279	chr17	13440075	13446545	*Smok2a*
WT1	chr17	13433078	13458279	chr17	13449144	13456071	*Smok2b*
WT2	chr1	88138955	88184803	chr1	88139681	88146719	*Ugt1a1*
WT2	chr4	136332370	136356223	chr4	136337748	136340537	*Tex46*
WT2	chr5	24639608	24655928	chr5	24643438	24650285	*Fastk*
WT2	chr5	24639608	24655928	chr5	24650456	24652852	*Tmub1*
WT2	chr5	25427846	25428588	chr5	25427846	25428151	*E130116L18Rik*
WT2	chr8	19297542	19301227	chr8	19297596	19300844	*Defb5*
WT2	chr8	115144223	115154275	chr8	115144826	115152586	*Clec3a*
WT2	chr9	108736926	108752150	chr9	108743687	108744631	*Tmem89*
WT2	chr17	13436747	13458908	chr17	13440075	13446545	*Smok2a*
WT2	chr17	13436747	13458908	chr17	13449144	13456071	*Smok2b*
WT2	chr19	5777890	5780417	chr19	5778115	5779681	*Fam89b*
WT2	chr19	47139224	47155304	chr19	47140048	47146203	*Calhm3*
WT3	chr1	88139117	88173315	chr1	88139681	88146719	*Ugt1a1*
WT3	chr8	93624612	93631124	chr8	93628035	93629039	*Capns2*
WT3	chr19	47137404	47150438	chr19	47140048	47146203	*Calhm3*
MCL1	chr2	24859577	24866010	chr2	24864129	24865110	*Mrpl41*
MCL1	chr2	27326957	27348407	chr2	27332336	27334233	*Brd3os*
MCL1	chr4	132028593	132031030	chr4	132029508	132030696	*Rab42*
MCL1	chr6	38085357	38093346	chr6	38086190	38092744	*Tmem213*
MCL1	chr7	126969975	126975384	chr7	126971709	126975222	*Zfp747*
MCL1	chr8	111782961	111785535	chr8	111782971	111784296	*Exosc6*
MCL1	chr10	75605971	75609940	chr10	75607064	75609248	*Ddt*
MCL1	chr13	23940875	23945433	chr13	23940996	23941392	*H4c2*
MCL1	chr13	23940875	23945433	chr13	23944675	23945213	*H4c1*
MCL1	chr17	27781671	27800171	chr17	27782547	27784730	*Smim29*
MCL1	chrX	94600343	94602643	chrX	94601710	94602409	*Pfn5*
MCL2	chr2	152403217	152412052	chr2	152404897	152411458	*Gm17416*
MCL2	chr10	128042950	128048431	chr10	128045777	128047676	*Spryd4*
MCL2	chr17	13442937	13458005	chr17	13449144	13456071	*Smok2b*
MCL2	chr2	25388004	25392108	chr2	25388663	25391731	*C8g*
MCL2	chr4	128998668	129007664	chr4	128999341	129005419	*Tmem54*
MCL2	chr5	115435039	115441508	chr5	115435169	115439058	*Dynll1*
MCL2	chr6	131662729	131666334	chr6	131663495	131664458	*Tas2r105*
MCL2	chr7	126612862	126616695	chr7	126614205	126616524	*Pagr1a*
MCL2	chr8	71915559	71919182	chr8	71917603	71918391	*Mrpl34*
MCL2	chr9	108215480	108218880	chr9	108216102	108217542	*Gpx1*
MCL2	chr10	82700868	82704799	chr10	82702460	82703764	*Eid3*

MCL2	chr11	72101167	72112390	chr11	72102550	72106418	*Med31*
MCL2	chr11	72101167	72112390	chr11	72105964	72109270	*4930563E22Rik*
MCL2	chr11	120652816	120655283	chr11	120653620	120655245	*Hmga1b*
MCL3	chr1	88138901	88184406	chr1	88139681	88146719	*Ugt1a1*
MCL3	chr1	173100266	173106057	chr1	173101949	173105344	*Mptx2*
MCL3	chr2	18000951	18002248	chr2	18001228	18001769	*H2al2a*
MCL3	chr2	152577438	152580878	chr2	152578171	152579330	*Id1*
MCL3	chr4	3870655	3874326	chr4	3870657	3872105	*Mos*
MCL3	chr4	88439944	88445775	chr4	88440262	88441011	*Ifnb1*
MCL3	chr4	118772679	118777852	chr4	118773721	118774662	*Or10ak8*
MCL3	chr4	138694111	138700136	chr4	138694423	138695676	*Rnf186*
MCL3	chr6	40470035	40475290	chr6	40470463	40471475	*Tas2r108*
MCL3	chr6	96141391	96143923	chr6	96141478	96143224	*Nup50l*
MCL3	chr6	132818659	132825642	chr6	132824105	132825106	*Tas2r123*
MCL3	chr7	104382129	104386668	chr7	104382592	104383557	*Or52n1*
MCL3	chr7	141710131	141711865	chr7	141710191	141710850	*Gm29735*
MCL3	chr8	19243910	19247174	chr8	19244373	19245228	*Defb14*
MCL3	chr11	99515502	99519621	chr11	99518050	99519064	*Krtap4-1*
MCL3	chr13	110487329	110493929	chr13	110489150	110493733	*Gapt*
MCL3	chr13	120776702	120780412	chr13	120778414	120779714	*Tcstv3*
MCL3	chr15	98377329	98383171	chr15	98378281	98380602	*Lalba*
MCL3	chr17	14168478	14172304	chr17	14168635	14170940	*Gm7168*
MCL3	chr18	36856558	36859977	chr18	36858120	36859851	*Cd14*
MCL3	chrX	72271872	72274647	chrX	72271897	72274401	*F8a*

Table S2. List of full genes present in eccDNA.

Patient	Age	Sex	Liver disease background	HCC tumor grade	Therapeutic action
Normal1	55	f	None; liver wedge resection (Seg. II) for neuroendocrine tumour of ileum (NET G1)	na	no chemotherapy before liver surgery
Normal2	36	m	None; right hemihepatectomy for manifestation of a solitary fibrous tumour (SFT)	na	no chemotherapy before liver surgery
Normal3	60	m	None; right hemihepatectomy for manifestation of a colorectal adenocarcinoma	na	no chemotherapy before liver surgery
Normal4	79	f	None; liver wedge resection (Seg. VI/VII) for manifestation of a colorectal adenocarcinoma	na	no chemotherapy before liver surgery
Normal5	57	f	None; liver wedge resection (Seg. IV) for manifestation of a colorectal adenocarcinoma	na	no chemotherapy before liver surgery
Normal6	30	f	None; liver biopsy in the context of live liver donation	na	no chemotherapy before liver biopsy
Normal7	74	m	liver wedge resection (Seg. VI) for intrahepatic cholangio-cellular adenocarcinoma (iCCA)	na	no chemotherapy before liver surgery
HCC1 & CLD1	73	m	Steatosis (anamnestically: no alcohol overconsumption)	G3	Surgery only: extended right hemihepatectomy
HCC2 & CLD2	64	m	Chronic HBV infection	G2	left hemihepatectomy (Seg. II & III); 2x TACE HCC Seg. VI; TACE HCC Seg. IVa; liver TPL; Therapy of chronic HBV infection
HCC3 & CLD3	63	m	Steatosis (BMI 29,6)	G2	atypical resection Seg. VII; right hemihepatectomy; resection of metastasis thoracic wall & post-op radiotherapy of thoracic wall
HCC4 & CLD4	73	m	Steatosis (BMI 29,7)	G3	Right hemihepatectomy & wedge resection Seg. III; start Sorafenib (because of diffuse liver involvement of HCC)
HCC5 & CLD5	86	f	no cirrhosis on histology: only minimal small droplet steatosis; anamnestically: no alcohol overconsumption (BMI 25.5)	G3	Right hemihepatectomy
HCC6 & CLD6	52	f	Steatosis: MASH, but very mild fibrosis (no cirrhosis) (BMI 38,4)	G3	left hemihepatectomy (Seg. II, III & IV)
HCC7 & CLD8	39	m	no cirrhosis; HCC fibrolamellar	G2	left hemihepatectomy & resection of lymph node metastasis retroperitoneal; Radiotherapy of lymph node metas mediastinum & retroperitoneal; start Sorafenib; included in Resource study (Bern): Regorafenib/Placebo

Table S3. Information on the patients from whom tissue was used in this study.

**Antibody**	**Company**	**Cat. #**	**Dilution**
Rabbit anti-MCL1	abcam	ab32087	1:1000
Mouse anti-β-Catenin	BD	610153	1:400
Mouse anti-γH2AX	BioLegend	613410	1:100
Rat anti-CldU	abcam	ab6326	1:400
Mouse anti-IdU	BD	347580	1:80
Mouse anti-ssDNA	DSHB	10805144	1:100
Rabbit anti-Ki67	abcam	ab16667	1:100
Rabbit anti-Cl. Caspase 3	Cell Signaling	9664S	1:100
Rat anti-F4/80	Biomedicals AG	T-2006	1:50
Rabbit anti-γH2AX	Novus	NB100-384	1:200
Rat anti-B220	Pharmingen	553084	1:4000
Rat anti-Ly6G	Pharmingen	551459	1:600
Rabbit anti-CD3	Thermo Fischer	MA1-90582	1:300
Rabbit anti-cGAS	Cell Signaling	31659S	1:100
Rabbit anti-STING	proteintech	19851-I-AP	1:1000

Table S4. List of antibodies used for IHC and IF

**Antibody**	**Company**	**Cat. #**	**Dilution**
Rabbit anti- MCL1	Rockland	600-401-394	1:1000
Rabbit anti-cGAS	Cell Signaling	31659S	1:1000
Rabbit anti-STING	proteintech	19851-I-AP	1:1000
Rabbit anti-GAPDH	Cell Signaling	5174S	1:1000
Rabbit anti-p-p65	Cell Signaling	3033L	1:1000
Rabbit anti-p65	Cell Signaling	3034S	1:1000
Rabbit anti-CD45	Cell Signaling	72787S	1:1000
Rabbit anti-H3	Cell Signaling	4499S	1:1000
Rabbit anti-Albumin	Cell Signaling	4929S	1:1000
Rabbit anti-p-IRF3	Cell Signaling	4947S	1:1000
Rabbit anti-TBK1	Cell Signaling	5483T	1:1000
Rabbit anti-TBK1	Invitrogen	PA5-105919	1:1000

Table S5. List of antibodies used for Western blotting

**Antibody**	**Company**	**Cat. #**	**Dilution**
CD11b-FITC	BioLegend	101205	1:100
CD11c-V450	BioLegend	117329	1:100
F4/80-PerCP	BioLegend	123125	1:100

Table S6. List of antibodies used for flow cytometry

**Name**	**Sequence**	**Supplier**
Cgas-F	CAGGCGTCTTCTTCAGTCATAC	Integrated DNA Technologies
Cgas-R	CTGGTCTACAGAGGGAGTTACA	Integrated DNA Technologies
Sting1-F	ACATTCCCTGCTCTCTGTTG	Integrated DNA Technologies
Sting1-R	TCCCTCTCGCCATCTACTT	Integrated DNA Technologies

Table S7. List of oligonucleotide primers used for qPCR

REFERENCES AND NOTES

1. H. Rumgay, M. Arnold, J. Ferlay, O. Lesi, C. J. Cabasag, J. Vignat, M. Laversanne, K. A. McGlynn, I. Soerjomataram, Global burden of primary liver cancer in 2020 and predictions to 2040. *J. Hepatol.* **77**, 1598–1606 (2022).

2. J. D. Yang, P. Hainaut, G. J. Gores, A. Amadou, A. Plymoth, L. R. Roberts, A global view of hepatocellular carcinoma: Trends, risk, prevention and management. *Nat. Rev. Gastroenterol. Hepatol.* **16**, 589–604 (2019).

3. P. Le, M. Tatar, S. Dasarathy, N. Alkhouri, W. H. Herman, G. B. Taksler, A. Deshpande, W. Ye, O. A. Adekunle, A. M. Cullough, M. B. Rothberg, Estimated burden of metabolic dysfunction-associated steatotic liver disease in US adults, 2020 to 2050. *JAMA Netw. Open* **8**, e2454707 (2025).

4. G. Feng, G. Targher, C. D. Byrne, Y. Yilmaz, V. Wai-Sun Wong, C. R. Adithya Lesmana, L. A. Adams, J. Boursier, G. Papatheodoridis, M. el-Kassas, N. Méndez-Sánchez, S. Sookoian, L. Castera, W. K. Chan, F. Ye, S. Treeprasertsuk, H. Cortez-Pinto, H. H. Yu, W. Kim, M. Romero-Gómez, A. Nakajima, K. M. Win, S. U. Kim, A. G. Holleboom, G. Sebastiani, P. Ocama, J. D. Ryan, M. Lupșor-Platon, H. Ghazinyan, M. al-Mahtab, S. Hamid, N. Perera, K. A. Alswat, Q. Pan, M. T. Long, V. Isakov, M. Mi, M. Arrese, A. J. Sanyal, S. K. Sarin, N. C. Leite, L. Valenti, P. N. Newsome, H. Hagström, S. Petta, H. Yki-Järvinen, J. M. Schattenberg, M. I. Castellanos Fernández, I. A. Leclercq, G. Aghayeva, A. N. Elzouki, A. Tumi, A. I. Sharara, A. Labidi, F. M. Sanai, K. Matar, M. al-Mattooq, M. W. Akroush, M. Benazzouz, N. Debzi, M. Alkhatry, S. Barakat, S. A. al-Busafi, J. Rwegasha, W. Yang, A. Adwoa, C. K. Opio, M. Sotoudeheian, Y. J. Wong, J. George, M. H. Zheng, Global burden of metabolic dysfunction-associated steatotic liver disease, 2010 to 2021. *JHEP Rep.* **7**, 101271 (2025).

5. M. Ringelhan, D. Pfister, T. O'Connor, E. Pikarsky, M. Heikenwalder, The immunology of hepatocellular carcinoma. *Nat. Immunol.* **19**, 222–32 (2018).

6. A. Weber, R. Boger, B. Vick, T. Urbanik, J. Haybaeck, S. Zoller, A. Teufel, P. H. Krammer, J. T. Opferman, P. R. Galle, M. Schuchmann, M. Heikenwalder, H. Schulze-Bergkamen, Hepatocyte-

specific deletion of the antiapoptotic protein myeloid cell leukemia-1 triggers proliferation and hepatocarcinogenesis in mice. *Hepatology* **51**, 1226–1236 (2010).

7. H. Nakagawa, A. Umemura, K. Taniguchi, J. Font-Burgada, D. Dhar, H. Ogata, Z. Zhong, M. A. Valasek, E. Seki, J. Hidalgo, K. Koike, R. J. Kaufman, M. Karin, ER stress cooperates with hypernutrition to trigger TNF-dependent spontaneous HCC development. *Cancer Cell* **26**, 331–343 (2014).

8. Y. Boege, M. Malehmir, M. E. Healy, K. Bettermann, A. Lorentzen, M. Vucur, A. K. Ahuja, F. Böhm, J. C. Mertens, Y. Shimizu, L. Frick, C. Remouchamps, K. Mutreja, T. Kähne, D. Sundaravinayagam, M. J. Wolf, H. Rehrauer, C. Koppe, T. Speicher, S. Padrissa-Altés, R. Maire, J. M. Schattenberg, J. S. Jeong, L. Liu, S. Zwirner, R. Boger, N. Hüser, R. J. Davis, B. Müllhaupt, H. Moch, H. Schulze-Bergkamen, P. A. Clavien, S. Werner, L. Borsig, S. A. Luther, P. J. Jost, R. Weinlich, K. Unger, A. Behrens, L. Hillert, C. Dillon, M. di Virgilio, D. Wallach, E. Dejardin, L. Zender, M. Naumann, H. Walczak, D. R. Green, M. Lopes, I. Lavrik, T. Luedde, M. Heikenwalder, A. Weber, A dual role of caspase-8 in triggering and sensing proliferation-associated DNA damage, a key determinant of liver cancer development. *Cancer Cell* **32**, 342–359.e10 (2017).

9. L. W. Thomas, C. Lam, S. W. Edwards, Mcl-1; The molecular regulation of protein function. *FEBS Lett.* **584**, 2981–2989 (2010).

10. D. A. Jackson, A. Pombo, Replicon clusters are stable units of chromosome structure: Evidence that nuclear organization contributes to the efficient activation and propagation of s phase in human cells. *J. Cell Biol.* **140**, 1285–1295 (1998).

11. C. J. Merrick, D. Jackson, J. F. X. Diffley, Visualization of altered replication dynamics after DNA damage in human cells. *J. Biol. Chem.* **279**, 20067–20075 (2004).

12. M. Di Bona, S. F. Bakhoum, Micronuclei and cancer. *Cancer Discov.* **14**, 214–226 (2024).

13. J. Kwon, S. F. Bakhoum, The cytosolic DNA-sensing cGAS-STING pathway in cancer. *Cancer Discov.* **10**, 26–39 (2020).

14. X. Luo, H. Li, L. Ma, J. Zhou, X. Guo, S. L. Woo, Y. Pei, L. R. Knight, M. Deveau, Y. Chen, X. Qian, X. Xiao, Q. Li, X. Chen, Y. Huo, K. McDaniel, H. Francis, S. Glaser, F. Meng, G. Alpini, C. Wu, Expression of STING is increased in liver tissues from patients with NAFLD and promotes macrophage-mediated hepatic inflammation and fibrosis in mice. *Gastroenterology* **155**, 1971–1984.e4 (2018).

15. M. K. Thomsen, R. Nandakumar, D. Stadler, A. Malo, R. M. Valls, F. Wang, L. S. Reinert, F. Dagnæs-Hansen, A. K. Hollensen, J. G. Mikkelsen, U. Protzer, S. R. Paludan, Lack of immunological DNA sensing in hepatocytes facilitates hepatitis B virus infection. *Hepatology* **64**, 746–59 (2016).

16. Y. Yu, Y. Liu, W. An, J. Song, Y. Zhang, X. Zhao, STING-mediated inflammation in Kupffer cells contributes to progression of nonalcoholic steatohepatitis. *J. Clin. Invest.* **129**, 546–55 (2019).

17. A. Ablasser, M. Goldeck, T. Cavlar, T. Deimling, G. Witte, I. Röhl, K. P. Hopfner, J. Ludwig, V. Hornung, cGAS produces a 2′-5′-linked cyclic dinucleotide second messenger that activates STING. *Nature* **498**, 380–384 (2013).

18. A. Ablasser, J. L. Schmid-Burgk, I. Hemmerling, G. L. Horvath, T. Schmidt, E. Latz, V. Hornung, Cell intrinsic immunity spreads to bystander cells via the intercellular transfer of cGAMP. *Nature* **503**, 530–534 (2013).

19. H. D. Moller, M. Mohiyuddin, I. Prada-Luengo, M. Reza Sailani, J. F. Halling, P. Plomgaard, L. Maretty, A. J. Hansen, M. P. Snyder, H. Pilegaard, H. Y. K. Lam, B. Regenberg, Circular DNA elements of chromosomal origin are common in healthy human somatic tissue. *Nat. Commun.* **9**, 1069 (2018).

20. Y. Wang, M. Wang, M. N. Djekidel, H. Chen, D. Liu, F. W. Alt, Y. Zhang, eccDNAs are apoptotic products with high innate immunostimulatory activity. *Nature* **599**, 308–314 (2021).

21. L. W. Dillon, P. Kumar, Y. Shibata, Y.-H. Wang, S. Willcox, J. D. Griffith, Y. Pommier, S. Takeda, A. Dutta, Production of extrachromosomal MicroDNAs is linked to mismatch repair pathways and transcriptional activity. *Cell Rep.* **11**, 1749–1759 (2015).

22. Y. Wang, M. Wang, Y. Zhang, Purification, full-length sequencing and genomic origin mapping of eccDNA. *Nat. Protoc.* **18**, 683–699 (2023).

23. G. Sankar, M. Karin, Missing pieces in the NF-κB puzzle. *Cell* **109**, S81–S96 (2002).

24. S. Luecke, A. Holleufer, M. H. Christensen, K. L. Jønsson, G. A. Boni, L. K. Sørensen, M. Johannsen, M. R. Jakobsen, R. Hartmann, S. R. Paludan, cGAS is activated by DNA in a length-dependent manner. *EMBO Rep.* **18**, 1707–1715 (2017).

25. Y. Liu, X. Chen, Y. Zhao, X. Y. Wang, Y. W. Luo, L. Chen, W. Wang, S. Zhong, M. Hu, Z. Dai, J. Jiang, X. Wang, H. Ji, X. X. Cheng, A. Zheng, J. Zuo, H. Liu, D. Ma, Z. Luo, F. Cao, S. Hu, A. L. Huang, K. F. Tang, Small cytosolic double-stranded DNA represses cyclic GMP-AMP synthase activation and induces autophagy. *Cell Rep.* **42**, 112852 (2023).

26. A. Henssen, I. MacArthur, R. Koche, H. Dorado-García, A. Henssen, Purification and sequencing of large circular DNA from human cells. *Protoc. Exch.*, 10.1038/protex.2019.006 (2019).

27. R. P. Koche, E. Rodriguez-Fos, K. Helmsauer, M. Burkert, I. C. MacArthur, J. Maag, R. Chamorro, N. Munoz-Perez, M. Puiggròs, H. Dorado Garcia, Y. Bei, C. Röefzaad, V. Bardinet, A. Szymansky, A. Winkler, T. Thole, N. Timme, K. Kasack, S. Fuchs, F. Klironomos, N. Thiessen, E. Blanc, K. Schmelz, A. Künkele, P. Hundsdörfer, C. Rosswog, J. Theissen, D. Beule, H. Deubzer, S. Sauer, J. Toedling, M. Fischer, F. Hertwig, R. F. Schwarz, A. Eggert, D. Torrents, J. H. Schulte, A. G. Henssen, Extrachromosomal circular DNA drives oncogenic genome remodeling in neuroblastoma. *Nat. Genet.* **52**, 29–34 (2020).

28. C. Z. Zhang, A. Spektor, H. Cornils, J. M. Francis, E. K. Jackson, S. Liu, M. Meyerson, D. Pellman, Chromothripsis from DNA damage in micronuclei. *Nature* **522**, 179–184 (2015).

29. P. J. Stephens, C. D. Greenman, B. Fu, F. Yang, G. R. Bignell, L. J. Mudie, E. D. Pleasance, K. W. Lau, D. Beare, L. A. Stebbings, S. McLaren, M. L. Lin, D. McBride, I. Varela, S. Nik-Zainal, C. Leroy, M. Jia, A. Menzies, A. P. Butler, J. W. Teague, M. A. Quail, J. Burton, H. Swerdlow, N. P. Carter, L. A. Morsberger, C. Iacobuzio-Donahue, G. A. Follows, A. R. Green, A. M. Flanagan, M. R. Stratton, P. A. Futreal, P. J. Campbell, Massive genomic rearrangement acquired in a single catastrophic event during cancer development. *Cell* **144**, 27–40 (2011).

30. E. Toufektchan, J. Maciejowski, Purification of micronuclei from cultured cells by flow cytometry. *STAR Protoc.* **2**, 100378 (2021).

31. K. Müller, H. Honcharova-Biletska, C. Koppe, M. Egger, L. K. Chan, A. T. Schneider, L. Küsgens, F. Böhm, Y. Boege, M. E. Healy, J. Schmitt, S. Comtesse, M. Castoldi, C. Preisinger, M. Szydlowska, E. Focaccia, N. T. Gaisa, S. H. Loosen, S. Jörs, F. Tacke, C. Roderburg, V. Keitel, J. G. Bode, P. Boor, R. J. Davis, T. Longerich, F. Geisler, M. Heikenwalder, A. Weber, M. Vucur, T. Luedde, JNK signaling prevents biliary cyst formation through a CASPASE-8-dependent function of RIPK1 during aging. *Proc. Natl. Acad. Sci. U.S.A.* **118**, e2007194118 (2021).

32. J. He, M. Gerstenlauer, L. K. Chan, F. Leithäuser, M. M. Yeh, T. Wirth, H. J. Maier, Block of NF-kB signaling accelerates MYC-driven hepatocellular carcinogenesis and modifies the tumor phenotype towards combined hepatocellular cholangiocarcinoma. *Cancer Lett.* **458**, 113–122 (2019).

33. M. J. Wolf, A. Adili, K. Piotrowitz, Z. Abdullah, Y. Boege, K. Stemmer, M. Ringelhan, N. Simonavicius, M. Egger, D. Wohlleber, A. Lorentzen, C. Einer, S. Schulz, T. Clavel, U. Protzer, C. Thiele, H. Zischka, H. Moch, M. Tschöp, A. V. Tumanov, D. Haller, K. Unger, M. Karin, M. Kopf, P. Knolle, A. Weber, M. Heikenwalder, Metabolic activation of intrahepatic CD8+ T cells and NKT cells causes nonalcoholic steatohepatitis and liver cancer via cross-talk with hepatocytes. *Cancer Cell* **26**, 549–564 (2014).

34. Z. Strizova, I. Benesova, R. Bartolini, R. Novysedlak, E. Cecrdlova, L. K. Foley, I. Striz, M1/M2 macrophages and their overlaps – Myth or reality? *Clin. Sci. (Lond.)* **137**, 1067–1093 (2023).

35. C. Wang, C. Ma, L. Gong, Y. Guo, K. Fu, Y. Zhang, H. Zhou, Y. Li, Macrophage polarization and its role in liver disease. *Front. Immunol.* **12**, 803037 (2021).

36. T. M. de Almeida, R. C. Leitão, J. D. Andrade, W. Beçak, F. J. Carrilho, S. Sonohara, Detection of micronuclei formation and nuclear anomalies in regenerative nodules of human cirrhotic livers and relationship to hepatocellular carcinoma. *Cancer Genet. Cytogenet.* **150**, 16–21 (2004).

37. H. Dungrawala, K. L. Rose, K. P. Bhat, K. N. Mohni, G. G. Glick, F. B. Couch, D. Cortez, The replication checkpoint prevents two types of fork collapse without regulating replisome stability. *Mol. Cell* **59**, 998–1010 (2015).

38. Y. Zhao, L. Yu, S. Zhang, X. Su, X. Zhou, Extrachromosomal circular DNA: Current status and future prospects. *eLife* **11**, e81412 (2022).

39. L. Mohr, E. Toufektchan, P. von Morgen, K. Chu, A. Kapoor, J. Maciejowski, ER-directed TREX1 limits cGAS activation at micronuclei. *Mol. Cell* **81**, 724–738.e9 (2021).

40. S. T. Sin, J. Deng, L. Ji, M. Yukawa, R. W. Chan, S. Volpi, A. Vaglio, P. Fenaroli, P. Bocca, S. H. Cheng, D. K. Wong, K. O. Lui, P. Jiang, K. C. A. Chan, R. W. Chiu, Y. M. D. Lo, Effects of nucleases on cell-free extrachromosomal circular DNA. *JCI Insight* **7**, e156070 (2022).

41. E. Pikarsky, R. M. Porat, I. Stein, R. Abramovitch, S. Amit, S. Kasem, E. Gutkovich-Pyest, S. Urieli-Shoval, E. Galun, Y. Ben-Neriah, NF-κB functions as a tumour promoter in inflammation-associated cancer. *Nature* **431**, 461–466 (2004).

42. Z. Dou, K. Ghosh, M. G. Vizioli, J. Zhu, P. Sen, K. J. Wangensteen, J. Simithy, Y. Lan, Y. Lin, Z. Zhou, B. C. Capell, C. Xu, M. Xu, J. E. Kieckhaefer, T. Jiang, M. Shoshkes-Carmel, K. M. A. A. Tanim, G. N. Barber, J. T. Seykora, S. E. Millar, K. H. Kaestner, B. A. Garcia, P. D. Adams, S. L. Berger, Cytoplasmic chromatin triggers inflammation in senescence and cancer. *Nature* **550**, 402–406 (2017).

43. S. Glück, B. Guey, M. F. Gulen, K. Wolter, T.-W. Kang, N. A. Schmacke, A. Bridgeman, J. Rehwinkel, L. Zender, A. Ablasser, Innate immune sensing of cytosolic chromatin fragments through cGAS promotes senescence. *Nat. Cell Biol.* **19**, 1061–1070 (2017).

44. H. Yang, H. Wang, J. Ren, Q. Chen, Z. J. Chen, cGAS is essential for cellular senescence. *Proc. Natl. Acad. Sci. U.S.A.* **114**, E4612–E4620 (2017).

45. H. Liu, H. Zhang, X. Wu, D. Ma, J. Wu, L. Wang, Y. Jiang, Y. Fei, C. Zhu, R. Tan, P. Jungblut, G. Pei, A. Dorhoi, Q. Yan, F. Zhang, R. Zheng, S. Liu, H. Liang, Z. Liu, H. Yang, J. Chen, P. Wang, T. Tang, W. Peng, Z. Hu, Z. Xu, X. Huang, J. Wang, H. Li, Y. Zhou, F. Liu, D. Yan, S. H. E. Kaufmann, C. Chen, Z. Mao, B. Ge, Nuclear cGAS suppresses DNA repair and promotes tumorigenesis. *Nature* **563**, 131–136 (2018).

46. C. Zierhut, N. Yamaguchi, M. Paredes, J. D. Luo, T. Carroll, H. Funabiki, The cytoplasmic DNA sensor cGAS promotes mitotic cell death. *Cell* **178**, 302–315.e23 (2019).

47. G. Dunphy, S. M. Flannery, J. F. Almine, D. J. Connolly, C. Paulus, K. L. Jønsson, M. R. Jakobsen, M. M. Nevels, A. G. Bowie, L. Unterholzner, Non-canonical activation of the DNA sensing adaptor STING by ATM and IFI16 mediates NF-κB signaling after nuclear DNA damage. *Mol. Cell* **71**, 745–760.e5 (2018).

48. M. Charni-Natan, I. Goldstein, Protocol for primary mouse hepatocyte isolation. *STAR Protoc.* **1**, 100086 (2020).

49. G. Toda, T. Yamauchi, T. Kadowaki, K. Ueki, Preparation and culture of bone marrow-derived macrophages from mice for functional analysis. *STAR Protoc.* **2**, 100246 (2021).
